# Widely targeted quantitative lipidomics and prognostic model reveal plasma lipid predictors for nasopharyngeal carcinoma

**DOI:** 10.1186/s12944-023-01830-2

**Published:** 2023-06-26

**Authors:** Xi Chen, Ying-xue Li, Xun Cao, Meng-yun Qiang, Chi-xiong Liang, Liang-ru Ke, Zhuo-chen Cai, Ying-ying Huang, Ze-jiang Zhan, Jia-yu Zhou, Ying Deng, Lu-lu Zhang, Hao-yang Huang, Xiang Li, Jing Mei, Guo-tong Xie, Xiang Guo, Xing Lv

**Affiliations:** 1 State Key Laboratory of Oncology in South China, Collaborative Innovation Center for Cancer Medicine, Guangdong Key Laboratory of Nasopharyngeal Carcinoma Diagnosis and Therapy, Sun Yat-Sen University Cancer Center, Guangzhou, 510060 China; 2 Department of Nasopharyngeal Carcinoma, Sun Yat-Sen University Cancer Center, Guangzhou, 510060 China; 3 Ping An Technology, Shenzhen, 518000 China; 4 Department of Intensive Care Unit, Sun Yat-Sen University Cancer Center, Guangzhou, 510060 China; 5 Department of Head and Neck Radiotherapy, the Cancer Hospitalof the, University of Chinese Academy of Sciences, Zhejiang Cancer Hospital, Institute of Basic Medicine and Cancer, Chinese Academy of Sciences , Hangzhou, 310022 China; 6 Department of Radiology, Sun Yat-Sen University Cancer Center, Guangzhou, 510060 China

**Keywords:** Lipidomics, Survival model, Lipid biomarkers, Immune and metabolic dysregulation, Nasopharyngeal carcinoma

## Abstract

**Background:**

Dysregulation of lipid metabolism is closely associated with cancer progression. The study aimed to establish a prognostic model to predict distant metastasis-free survival (DMFS) in patients with nasopharyngeal carcinoma (NPC), based on lipidomics.

**Methods:**

The plasma lipid profiles of 179 patients with locoregionally advanced NPC (LANPC) were measured and quantified using widely targeted quantitative lipidomics. Then, patients were randomly split into the training (125 patients, 69.8%) and validation (54 patients, 30.2%) sets. To identify distant metastasis-associated lipids, univariate Cox regression was applied to the training set (*P* < 0.05). A deep survival method called DeepSurv was employed to develop a proposed model based on significant lipid species (*P* < 0.01) and clinical biomarkers to predict DMFS. Concordance index and receiver operating curve analyses were performed to assess model effectiveness. The study also explored the potential role of lipid alterations in the prognosis of NPC.

**Results:**

Forty lipids were recognized as distant metastasis-associated (*P* < 0.05) by univariate Cox regression. The concordance indices of the proposed model were 0.764 (95% confidence interval (CI), 0.682–0.846) and 0.760 (95% CI, 0.649–0.871) in the training and validation sets, respectively. High-risk patients had poorer 5-year DMFS compared with low-risk patients (Hazard ratio, 26.18; 95% CI, 3.52–194.80; *P* < 0.0001). Moreover, the six lipids were significantly correlated with immunity- and inflammation-associated biomarkers and were mainly enriched in metabolic pathways.

**Conclusions:**

Widely targeted quantitative lipidomics reveals plasma lipid predictors for LANPC, the prognostic model based on that demonstrated superior performance in predicting metastasis in LANPC patients.

**Supplementary Information:**

The online version contains supplementary material available at 10.1186/s12944-023-01830-2.

## Background

With an extremely unbalanced geographical distribution, over 70% of new diagnosed cases with nasopharyngeal carcinoma (NPC) are in Southern China and Southeast Asia [1, 2]. Over 70% cases are diagnosed with locoregionally advanced NPC (LANPC), with high risk of disease progression and unfavorable prognosis [3, 4]. As a result, patients with LANPC are recommended chemoradiotherapy by the National Comprehensive Cancer Network guidelines. However, inconsistent therapeutic effects have been reported in these patients [5]. Therefore, it is essential to understand the heterogeneous indicators that might affect the prognosis of NPC and provide insights into potential therapeutic targets.

Lipid species are actively involved in energy metabolism and cell signaling, dysregulation of which disturbs the progression states of diseases, including cancers [6, 7]. Altered supply of fatty acids (FAs) and cholesterol have emerged as unfavorable hallmarks that drove key oncogenic processes in cancers [8, 9]. This alteration has been associated with the increasing need for lipids as substrate and fuel to support the proliferation and metastasis of cancer cells [10, 11]. The rapid development of powerful means for studying lipids weaponized lipidomics in malignancy biology [9]. As some studies of liquid biopsies have shown, subsequent alterations in lipid metabolism have been linked to the onset, prognosis, or therapy response of cancer [12–14]. Studies have explored the relationship between altered lipid profiles and the initiation and progression of cancers, including liver cancers [15], medulloblastoma [16], colorectal cancers [17], breast cancers [14, 18], and lung cancers [19]. A previous study indicated that body mass index (BMI) was a survival predictor for patients with LANPC; low (< 18 kg*/m* ^*2*^) and high BMI values (> 24 kg*/m* ^*2*^), especially BMI values > 27 kg*/m* ^*2*^, increased the risk of NPC progression [20]. These results suggest that changes in lipid composition are potential prognostic indicators for NPC. The emergence of effective tools, including liquid chromatography (LC)–tandem mass spectrometry (MS/MS), will help to explored the promising role of fluctuate lipid metabolism in the progression of NPC.

This study aimed to establish a survival model to illuminate the relationship between lipid profiles and the clinical outcomes of patients with LANPC, including distant metastasis and disease progression. By utilizing widely targeted quantitative lipidomics, the exact quantitative plasma lipid profiles of 179 patients with LANPC were determined. Based on quantitative lipid profiles and traditional risk factors, a promising predictive model was established using the deep survival method called DeepSurv [21]. Patients with high risk scores calculated by the model had a poorer prognosis than those with low risk scores. The study further explored the mechanism underlying lipid alteration-mediated prognostic deterioration of NPC. Altogether, the study uncovered some promising lipid predictors for the prognosis of patients with LANPC.

## Patients and methods

### Study design

The study was designed to explore plasma lipidomic biomarkers predictive of survival in patients with LANPC. From December 2013 to June 2015, 179 patients with NPC treated in Sun Yat-sen University Cancer Center were included if they were (i) histologically confirmed LANPC; (ii) underwent concurrent chemoradiotherapy (CCRT) ± induction chemotherapy (ICT), and radiotherapy specifically reference to intensity-modulated radiotherapy (IMRT); (iii) BMI between 18.0 and 24.0; (iv) with no history of diabetes or cardiovascular disease; and (v) aged 18–70 years regardless of sex. Patients with a history of other malignancies, presence of primary distant metastasis, crucial organ dysfunction, and ineffective follow-up were excluded. All the patients were restaged according to the American Joint Committee on Cancer manual (8th edition).

### Study endpoints, treatment and follow-up

Distant metastasis-free survival (DMFS) was the primary endpoint and progression-free survival (PFS) and overall survival (OS) were the secondary endpoints of this study, which have been well defined previously [20]. The treatment and follow-up of patients with LANPC are described in more detail in Supplementary Materials.

### Lipid profiling analyses

Plasma samples were collected before the treatment. Based on Ultra Performance Liquid Chromatography (UPLC) and MS/MS, a method called widely targeted lipidomics was used to measure the lipid profiles. First, lipids were extracted from 50 μL plasma and dried subsequently. Second, two core systems named ExionLCTM AD (https://sciex.com.cn/) and QTRAP® 6500 + (https://sciex.com.cn/) were utilized to execute lipidomic analyses. Finally, absolute quantification of individual lipids from different classes was generated using respective internal standards. Additional details, including plasma sample collection, lipid metabolites analysis, LC and MS, and qualitative and quantitative evaluation on lipid profiles, are provided in Supplementary Materials and Supplementary Tables 1 and 2.

### Statistical analysis

For each patient, information on sociodemographic and clinical characteristics (with age, sex, BMI, tumor (T) stage, lymph node (N) stage, clinical stage, routine blood and biochemical parameters before treatment, initial Epstein–Barr virus (EBV) DNA concentration, and treatment regimens included) were collected. As a skewed distribution with large spans, the original continuous value of the EBV DNA concentration was transformed into categorical variables based on each tenfold change (< 1,000, 1,000–9,999, 10,000–99,999, and ≥ 100,000) [22].

A deep learning survival model based on lipids and clinical biomarkers was developed in the study. Prior to statistical analysis, lipid data were scaled by the standard deviation and mean for the sake of the modeling procedure. First, 125 (69.8%) and 54 (30.2%) patients were randomly split into the training and validation sets. The random method used have been well described previously [20]. Second, to recognize distant metastasis-associated lipids, the univariate Cox regression analysis was applied, and hazard ratios (HRs) and 95% confidence intervals (CIs) were estimated for the training set. Significant lipid species (*P* < 0.01) were further incorporated into the deep survival model. As age, sex, cancer stages, and EBV DNA are acknowledged as clinical predictors of NPC prognosis, these clinical biomarkers were also be incorporated into the predictive model. Third, a survival model built by DeepSurv was employed, with the status and time of the primary endpoint as the target outcome. The importance scores of features in the DeepSurv model were estimated by calculating the decrease in the model performance concordance index (C-index) [23] when the feature information was destroyed. Specifically, for each feature vector j, its values were permuted in the original data matrix A to generate a new data matrix, B. This broke the association between feature j and the true outcome, y. A new C-index based on the new matrix B was then calculated. The change in the C-index from matrix A to matrix B (${{\Delta C}_{j}= Cindex}_{B,j}-{Cindex}_{A,j}$) indicates the importance of feature j. The procedure was repeated 1000 times and defined the $\frac{1}{1000}\sum_{n=1}^{1000}{\Delta C}_{j,n}$ as the feature importance of feature j. To split patients into the “low-risk” and “high-risk” groups, the cutoff value was obtained according to the prediction by the proposed model on the training cohort by utilizing the “ctree” function in the R package “partykit” [24, 25], a method of recursive partitioning analysis. To further evaluate the prognostic effect of lipid signatures, a baseline model based only on clinical biomarkers, including age, sex, cancer stages, and EBV DNA, was constructed using the same modeling algorithm as the proposed model.

The predictive performance of the proposed model was then evaluated from different perspectives: C-index for assessing the discrimination ability [23]; the Hosmer–Lemeshow Goodness of Fit (GOF) Test [26] for evaluating the calibration of the model; receiver operating characteristic (ROC) curve analysis and the area under the ROC curve (AUC) for estimating sensitivity and specificity; and Kaplan–Meier plots and log-rank tests for comparing the survival rates of the risk groups. Robustness of the models constructed to predict the secondary endpoints was also assessed by utilizing the same algorithm to develop independent survival models based on the same lipid and clinical biomarkers in predicting PFS and OS. All metrics were calculated in the training cohort and verified in the validation cohort.

To seek information on potential effects of lipid alterations on NPC prognosis, the Spearman correlation between plasma lipids and clinical indices was investigated, which was implemented using the cor.test function in the stats package of R. Subsequently, the website tool MetaboAnalyst (version 5.0, https://www.metaboanalyst.ca/MetaboAnalyst/ModuleView.xhtml) was utilized to execute Kyoto Encyclopedia of Genes and Genomes (KEGG) pathway analysis to explore the underlying function of these differential lipid molecules.

Open-source libraries in Python (version 3.4.3) were utilized to practice Deep survival techniques. R (version 3.5.2) was utilized to practice other statistical analyses, and two-sided *P* < 0.05 was considered statistically significant.

## Results

### Baseline characteristics and follow-up

**Fig. 1:**
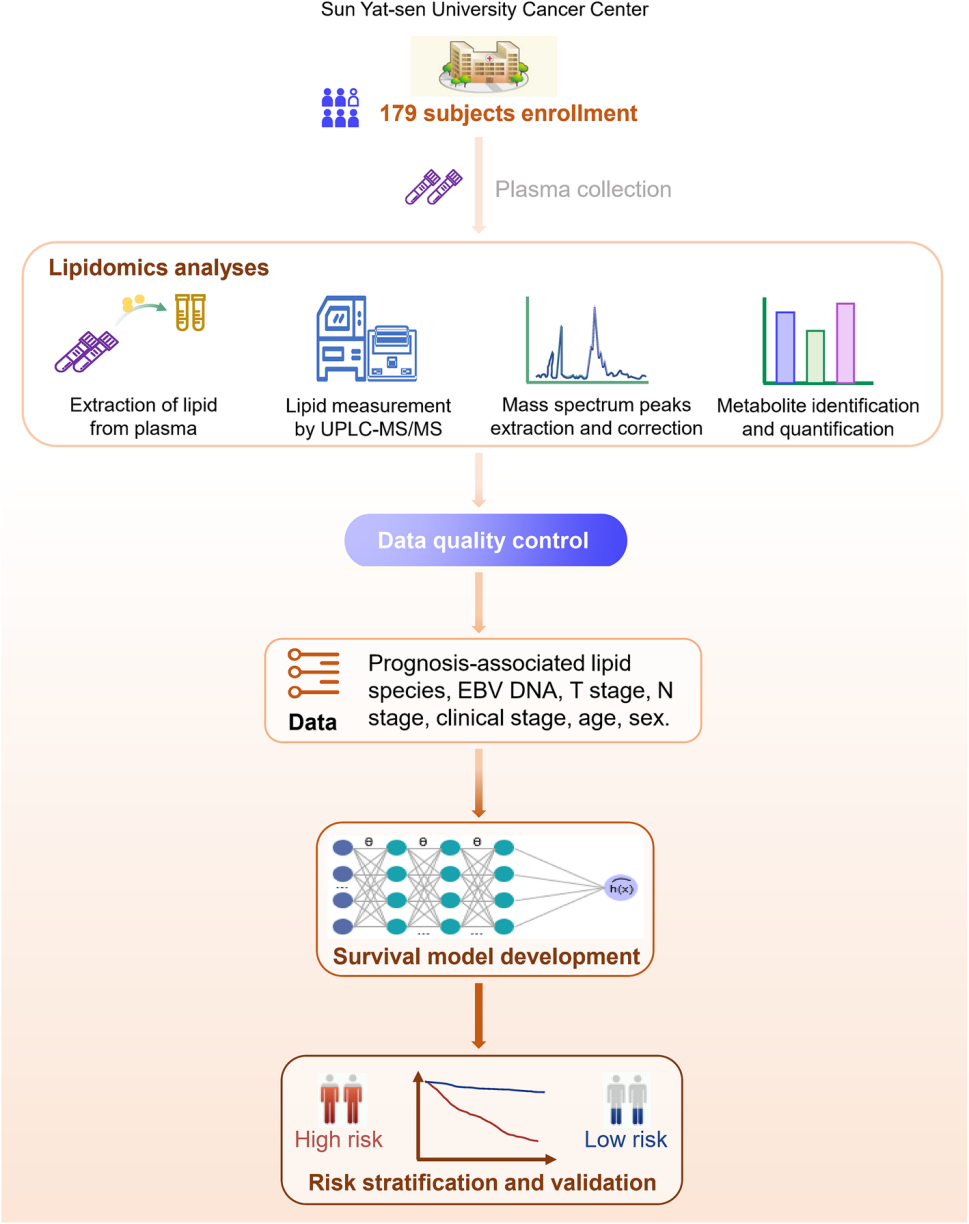
Flow chart of the study. Abbreviations: UPLC-MS/MS, Ultra Performance Liquid Chromatography and Tandem Mass Spectrometry; EBV, Epstein-Barr virus; T, tumor; N, lymph node

Between December 2013 and June 2015, 179 patients with NPC were included in this study. The flow chart is illustrated in Fig. 1. The mean age of the 179 patients was 45.7 years (SD [standard deviation], 12.1 years), and they were mostly male (131 patients; 73.2%). Of the 179 patients, 71 (39.7%) received CCRT and 108 (60.3%) received ICT plus CCRT. By the cutoff date of November 30, 2020, the median follow-up time was 70.6 months (IQR [interquartile range], 67.7–73.2 months). At the last follow-up, 39 (21.8%) patients developed distant metastases. Table 1 demonstrates the baseline characteristics of patients based on the status of the primary and secondary endpoints.

**Table 1:** Baseline characteristics based on the status of the primary and secondary endpoints

Value N (%) or mean ± SD	Distant metastasis			Progression			Death		
	**Control (** ***n*** ** = 140)**	**Case (** ***n*** ** = 39)**	***P*** ** value** ^**a**^	**Control (** ***n*** ** = 125)**	**Case (** ***n*** ** = 54)**	***P*** ** value** ^a^	**Control (** ***n*** ** = 162)**	**Case (** ***n*** ** = 17)**	***P*** ** value** ^a^
Age (yr)	45.2 ± 12.5	47.3 ± 10.6	0.313	45.0 ± 12.8	47.1 ± 10.2	0.249	45.6 ± 12.2	45.9 ± 11.9	0.923
Sex			0.106			0.028			0.566
Female	42 (30.0)	6 (15.4)		40 (32.0)	8 (14.8)		45 (27.8)	3 (17.6)	
Male	98 (70.0)	33 (84.6)		85 (68.0)	46 (85.2)		117 (72.2)	14 (82.4)	
Tumor stage			0.027			0.002			0.071
T1	8 (5.7)	0 (0)		8 (6.4)	0 (0)		8 (4.9)	0 (0)	
T2	13 (9.3)	2 (5.1)		12 (9.6)	3 (5.6)		14 (8.6)	1 (5.8)	
T3	94 (67.1)	22 (56.4)		86 (68.8)	30 (55.6)		108 (66.7)	8 (47.1)	
T4	25 (17.9)	15 (38.5)		19 (15.2)	21 (38.8)		32 (19.8)	8 (47.1)	
Node stage			0.007			0.001			0.671
N0	18 (12.9)	0 (0)		17 (13.6)	1 (1.9)		17 (10.5)	1 (5.9)	
N1	65 (46.4)	12 (30.8)		61 (48.8)	16 (29.6)		71 (43.8)	6 (35.3)	
N2	34 (24.3)	15 (38.5)		29 (23.2)	20 (37.0)		44 (27.2)	5 (29.4)	
N3	23 (16.4)	12 (30.7)		18 (14.4)	17 (31.5)		30 (18.5)	5 (29.4)	
Clinical stage			0.002			< 0.001			0.025
III	97 (69.3)	16 (41.0)		92 (73.6)	21 (38.9)		107 (66.0)	6 (35.3)	
IV	43 (30.7)	23 (59.0)		33 (26.4)	33 (61.1)		55 (34.0)	11 (64.7)	
BMI (kg/m2)	21.4 ± 1.5	21.9 ± 1.2	0.064	21.4 ± 1.5	21.7 ± 1.3	0.237	21.5 ± 1.5	21.5 ± 1.3	0.924
CHO (mmol/L)	4.9 ± 1.0	4.8 ± 0.8	0.626	4.9 ± 1.0	4.9 ± 0.9	0.575	4.9 ± 0.9	4.9 ± 1.3	0.952
TG (mmol/L)	1.3 ± 0.8	1.2 ± 0.6	0.840	1.2 ± 0.8	1.3 ± 0.6	0.530	1.3 ± 0.7	1.2 ± 0.6	0.625
HDL (mmol/L)	1.3 ± 0.3	1.4 ± 0.3	0.540	1.3 ± 0.3	1.3 ± 0.3	0.895	1.3 ± 0.3	1.4 ± 0.4	0.677
LDL (mmol/L)	3.0 ± 0.9	3.0 ± 0.8	0.588	3.0 ± 0.8	3.1 ± 0.9	0.475	3.0 ± 0.8	3.2 ± 1.4	0.682
WBC (10^9^/L)	7.1 ± 2.0	6.8 ± 1.6	0.424	7.0 ± 1.9	7.1 ± 2.0	0.769	7.0 ± 1.9	6.7 ± 2.4	0.633
Neutrophil (10^9^/L)	4.6 ± 1.7	4.0 ± 1.1	0.018	4.5 ± 1.6	4.4 ± 1.7	0.629	4.5 ± 1.6	4.0 ± 2.1	0.373
Lymphocyte (10^9^/L)	1.8 ± 0.6	2.1 ± 0.7	0.016	1.8 ± 0.6	2.0 ± 0.6	0.056	1.8 ± 0.6	2.0 ± 0.7	0.423
CRP (mg/L)	4.0 ± 7.1	3.7 ± 6.2	0.810	3.8 ± 6.5	4.3 ± 8.0	0.672	3.6 ± 6.1	7.0 ± 12.2	0.281
LDH (U/L)	180.2 ± 47.2	198.8 ± 78.8	0.168	178.8 ± 43.3	196.9 ± 76.7	0.108	183.5 ± 55.3	191.7 ± 62.7	0.608
EBV DNA (copies/ml)			0.064			0.193			0.244
< 1000	58 (41.4)	14 (35.9)		54 (43.2)	18 (33.3)		67 (41.4)	5 (29.4)	
1000–9999	47 (33.6)	9 (23.1)		41 (32.8)	15 (27.8)		52 (32.1)	4 (23.5)	
10,000–99,999	29 (20.7)	10 (25.6)		24 (19.2)	15 (27.8)		32 (19.8)	7 (41.2)	
≥ 100,000	6 (4.3)	6 (15.4)		6 (4.8)	6 (11.1)		11 (6.7)	1 (5.9)	
Treatment			0.703			0.488			0.013
CCRT	54 (38.6)	17 (43.6)		47 (37.6)	24 (44.4)		59 (36.4)	12 (70.6)	
ICT + CCRT	86 (61.4)	22 (56.4)		78 (62.4)	30 (55.6)		103 (63.6)	5 (29.4)	
Targeted therapy^b^	24 (17.1)	3 (7.7)	0.228	21(16.8)	6 (11.1)	0.454	26(16.0)	1 (5.9)	0.476

*BMI* Body mass index, *CHO* Cholesterol, *TG* Triglyceride, *HDL* High-density lipoprotein cholesterol, *LDL* Low-density lipoprotein cholesterol, *WBC* White blood cell, *CRP* C-reactive protein, *LDH* Lactate dehydrogenase, *EBV* Epstein-Barr virus, *CCRT* Concurrent chemoradiotherapy, *ICT* Induction chemotherapy

^a^ *P* values were calculated by the Chi-square test for categorical variables and the Student's T test for continuous variables

^b^Targeted therapy was defined as treatment with Cetuximab or Nimotuzumab

### Identification and quantification of plasma lipid profiles

In total, 665 plasma lipid metabolites consisting of 27 lipid classes and subclasses from 179 patients with NPC were annotated and quantified using lipidomics analysis (Supplementary Fig. 1). These lipid subclasses mainly included bile acid (BA), eicosanoid, free fatty acid (FFA), lysophosphatidylglycerol (LPG), lysophosphatidylinositol (LPI), lysophosphatidylserine (LPS), phosphatidylinositol (PI), ceramide-1-phosphate (CerP), acylcarnitine (CAR), cholesterol (Cho), cholesteryl ester (CE), sphingosine (SPH), ceramide (Cer), coenzyme Q (CoQ), diacylglycerol (DG), hexosylceramide (HexCer), lysophophatidylcholine (LPC), alkyl-lysophophatidylcholine (LPC-O), lysophosphatidylethanolamine (LPE), alkenyl-lysophosphatidylethanolamine (LPE-P), phosphatidylcholine (PC), alkylglycerophosphocholine (PC-O), phosphatidylethanolamine (PE), alkenylglycerophosphoethanolamine (PE-P), phosphatidylserine (PS), sphingomyelin (SM), and triacylglycerol (TG). Supplementary Table 3 demonstrates the details of the 655 lipid species annotated in the lipidomics analysis. To determine the signal stability of the mass spectrum, the overlapped total ion chromatogram of the plasma mixtures (quality control samples) is shown in Supplementary Fig. 2. The retention time and peak strength of the quality control samples were consistent, indicating the steady mass spectrum signal during sample analysis.

### Improvement of the survival model performance by prognostic lipid species

**Fig. 2:**
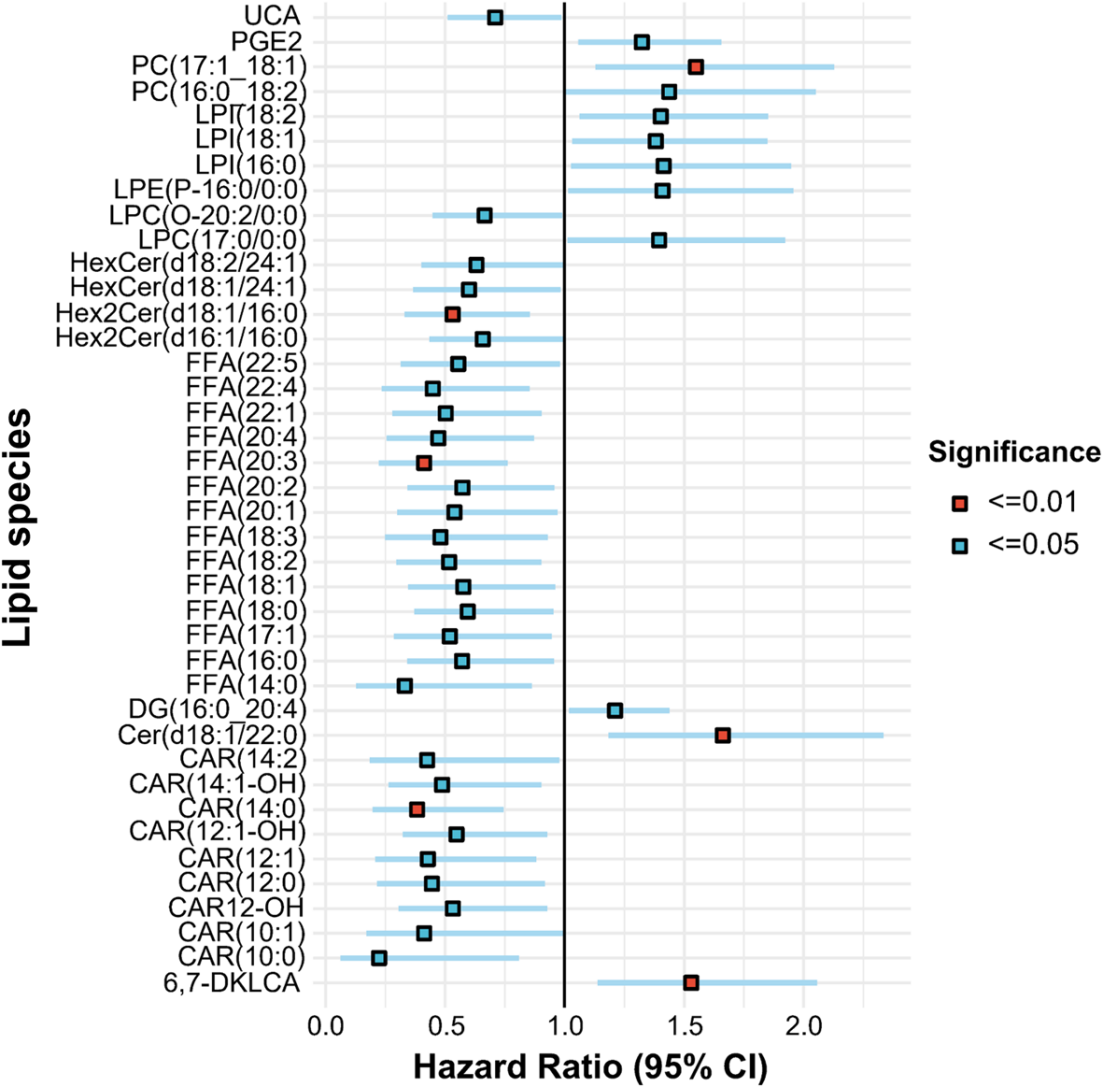
Hazard ratio (95% CI) for lipid species. Hazard ratios were obtained from univariate Cox regression analysis after adjustment for covariates including age, sex, BMI, T stage, N stage, clinical stage, Epstein-Barr virus DNA, CHO, TG, HDL, and LDL. For univariate Cox regression on the training set, lipid concentrations were mean centred and scaled by the standard deviation. Abbreviations: UCA, Ursocholic acid; PG, prostaglandin; PC, phosphatidylcholine; LPI, lysophosphatidylinositol; LPE, lysophosphatidylethanolamine; LPC, lysophophatidylcholine; HexCer, hexosylceramide; FFA, free fatty acid; DG, diacylglycerol; Cer, ceramide; CAR, acylcarnitine; DKLCA, diketolithocholic acid

Before building the survival model, patients were randomly split into the training (125 patients; 69.8%) and validation (54 patients; 30.2%) sets. The demographic and baseline characteristics based on the two data sets are demonstrated in Supplementary Table 4. After adjustment for covariates (with age, sex, BMI, cancer stages, EBV DNA, CHO, TG, HDL, and LDL included), 40 of the 655 lipid species were identified as distant metastasis-associated lipids by univariate Cox regression (*P* < 0.05) in the training set (Fig. 2 and Supplementary Table 5). In addition, Cer(d18:1/22:0) (HR, 1.66; 95% CI, 1.18–2.33; *P* = 0.003), FFA(20:3) (HR, 0.41; 95% CI, 0.22–0.76; *P* = 0.005), CAR(14:0) (HR, 0.38; 95% CI, 0.20–0.75; *P* = 0.005), 6,7-diketolithocholic acid (DKLCA) (HR, 1.53; 95% CI, 1.14–2.06; *P* = 0.005), PC(17:1_18:1) (HR, 1.55; 95% CI, 1.13–2.13; *P* = 0.007), and dihexosylceramide (Hex2Cer)(d18:1/16:0) (HR, 0.53; 95% CI, 0.33–0.85; *P* = 0.009)) were further incorporated into the survival model as predictive lipid species (*P* < 0.01).

**Fig. 3:**
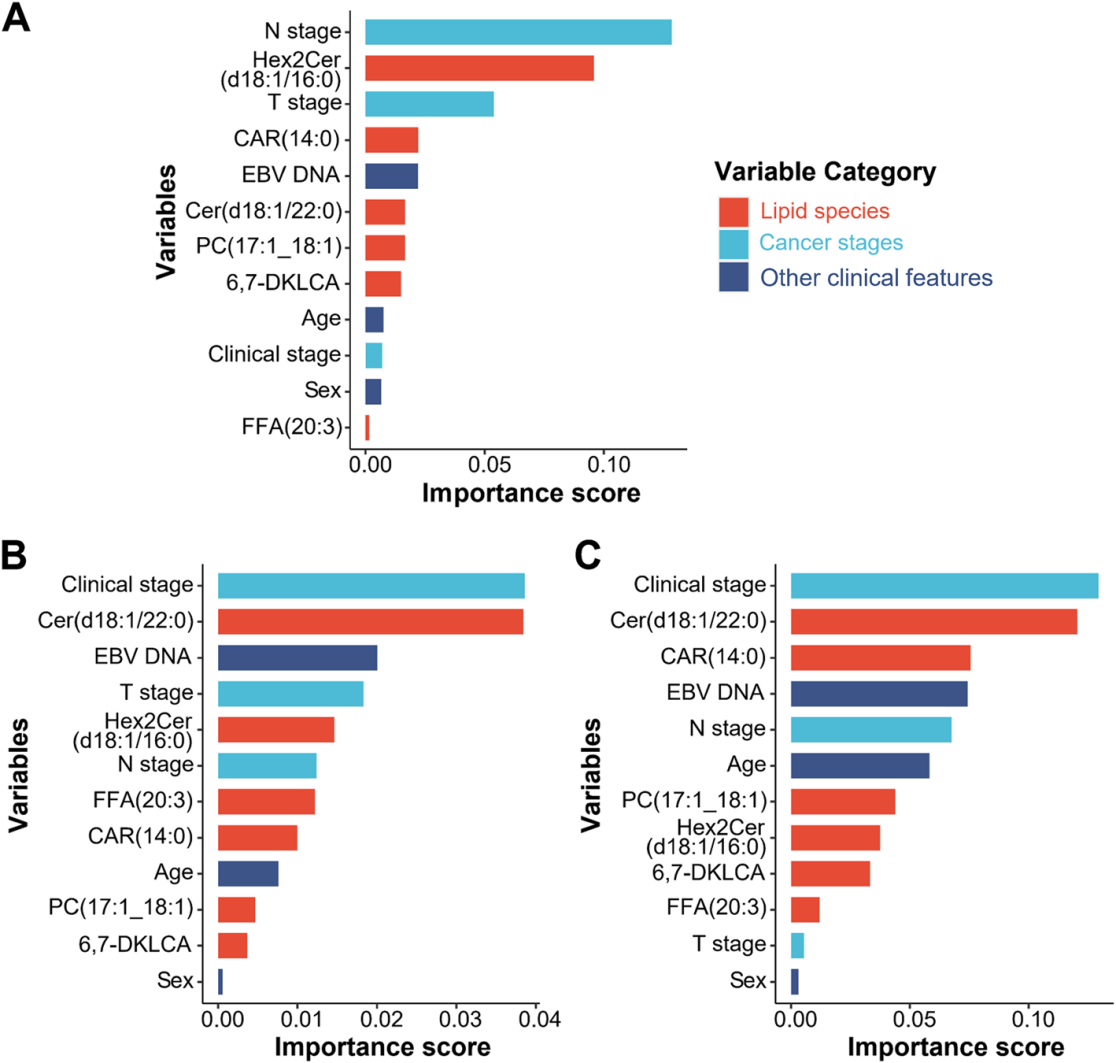
Variable importance in the proposed model based on lipid and clinical biomarkers. The feature importance score in predicting DMFS (**A**), PFS (**B**) and OS (**C**) was output in the DeepSurv model representing the decrease in the concordance index when the feature’s information was destroyed. The color of the bar represents the variable category. Abbreviations: DMFS, distant metastasis-free survival; PFS, progression-free survival; OS, overall survival; N, node stage; T, tumor stage; Hex2Cer, dihexosylceramide; CAR, acylcarnitine; EBV, Epstein-Barr virus; Cer, ceramides; PC, phosphatidylcholines; FFA, free fatty acid; DKLCA, diketolithocholic acid

The C-index values of the proposed prognostic model comprised of lipid and clinical biomarkers were 0.764 (95% CI, 0.682–0.846) and 0.760 (95% CI, 0.649–0.871) in the two data sets, respectively. According to the feature importance scores, Hex2Cer(d18:1/16:0), CAR(14:0), and Cer(d18:1/22:0), together with N stage, T stage, and EBV DNA, were the top six predictive variables of the proposed survival model (Fig. 3A), suggesting that Hex2Cer(d18:1/16:0), CAR(14:0), and Cer(d18:1/22:0) played important roles in predicting DMFS. The C-index values of the baseline survival model based only on clinical biomarkers were also calculated, with 0.718 (95% CI, 0.591–0.845) in the training set and 0.672 (95% CI, 0.511–0.833) in validation sets being detected respectively, indicating the outstanding predictive performance of lipid biomarkers. The *p* values of the calibration of the model were 0.700 and 0.803 in the training and validation sets in predicting DMFS. These insignificant *p*-values represented a good fit of the predicted results. As presented in Fig. 3B and C and Supplementary Table 6, lipid biomarkers also remained strong prognostic factors for predicting PFS and OS. ROC curve and AUC analyses were then implemented to compare the two models, which showed that the proposed model outperformed the baseline model in the perspectives of sensitivity and specificity (Table 2).

**Table 2:** The 5-year and 3-year AUCs of the proposed model and baseline model in the training and validation sets

Clinical outcome	Training set		Validation set	
	**AUC (95% CI) of the proposed model** ^a^	**AUC (95% CI) of the baseline model** ^b^	**AUC (95% CI) of the proposed model** ^a^	**AUC (95% CI) of the baseline model** ^b^
DMFS				
5-year	0.797 (0.719–0.874)	0.739 (0.636–0.841)	0.800 (0.696–0.904)	0.691 (0.559–0.823)
3-year	0.787 (0.706–0.868)	0.734 (0.625–0.844)	0.786 (0.678–0.895)	0.698 (0.560–0.835)
PFS				
5-year	0.795 (0.700–0.891)	0.707 (0.594–0.82)	0.802 (0.687–0.917)	0.698 (0.557–0.838)
3-year	0.766 (0.66–0.873)	0.708 (0.592–0.824)	0.791 (0.669–0.912)	0.698 (0.549–0.846)
OS				
5-year	0.768 (0.598–0.939)	0.699 (0.615–0.783)	0.739 (0.551–0.926)	0.65 (0.446–0.854)
3-year^c^	-	-	-	-

*DMFS* Distant metastasis-free survival, *PFS* Progression-free survival, *OS* Overall survival, *AUC* Area under the receiver operating characteristic curve, *CI* Confidence interval

^a^The proposed model was developed based on lipid and clinical biomarkers

^b^The baseline model was developed based on only clinical biomarkers

^c^AUCs of the survival models in predicting 3-year OS were not available by reason of only 1 patient dead within 3 years in the training set

**Fig. 4:**
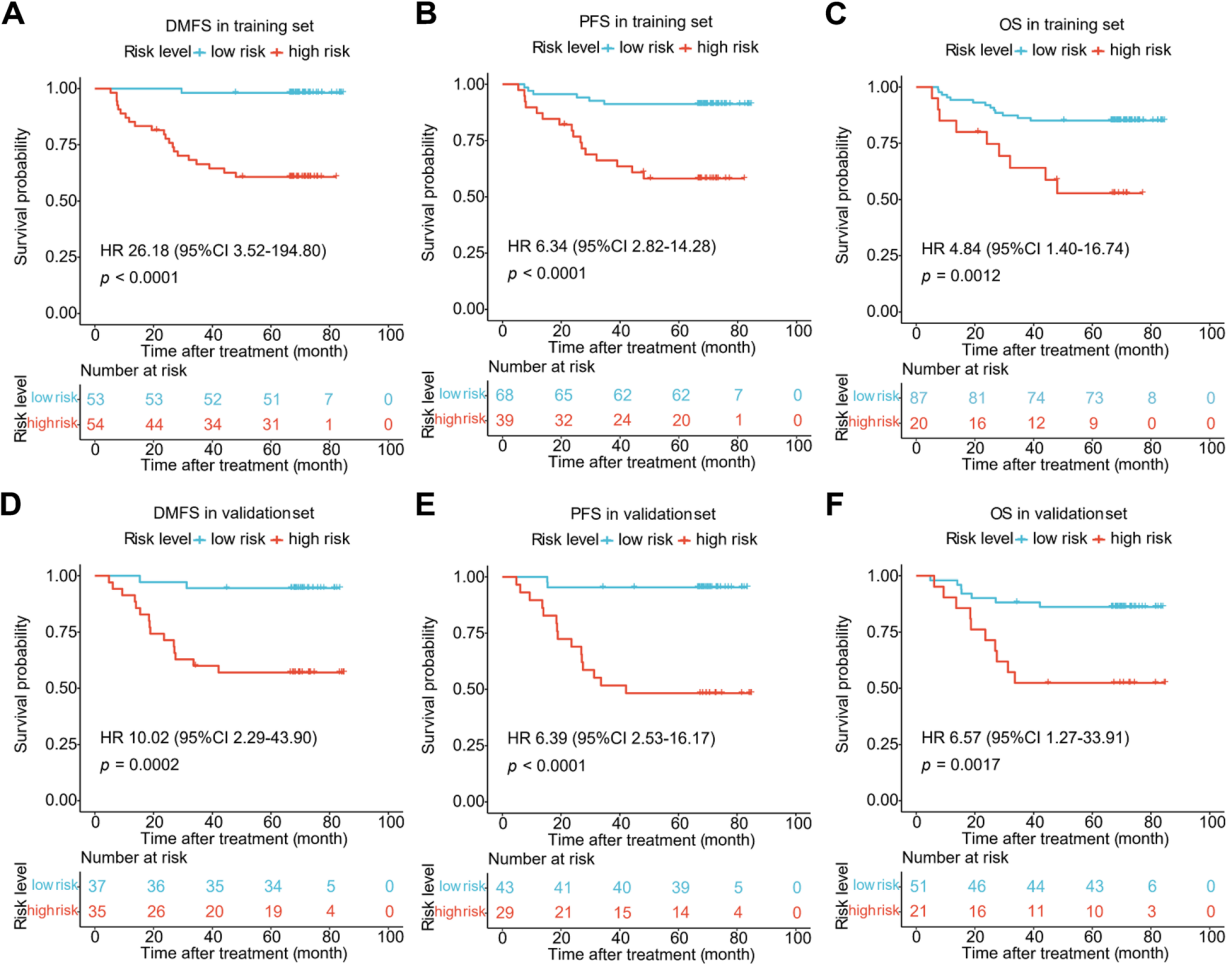
Kaplan-Meier curves of DMFS, PFS and OS stratified by low- and high-risk group. **A** DMFS in the training set. **B** PFS in the training set. **C** OS in the training set. **D** DMFS in the validation set. **E** PFS in the validation set. **F** OS in the validation set. *P*-values were calculated by the log-rank test. Hazard ratios were obtained from the univariate Cox regression analysis. Abbreviations: DMFS, distant metastasis-free survival; PFS, progression-free survival; OS, overall survival

Based on the optimal cutoff in predicting DMFS found in the training set, 53 (49.5%) and 54 (50.5%) patients were detected as low- and high-risk ones, respectively. The 5-year DMFS rate was 60.6% (95% CI, 48.8–75.3) for the high-risk patients and 98.1% (95% CI, 94.5–100) for the low-risk patients (HR, 26.18; 95% CI, 3.52–194.80; *P* < 0.0001; Fig. 4A). Compared with low-risk patients, poorer PFS (79.5% vs. 97.1%; HR, 6.34, 95% CI, 2.82–14.28; *P* < 0.0001; Fig. 4B) and OS (75.0% vs. 94.3%; HR, 4.84; 95% CI, 1.40–16.74; *P* = 0.0012; Fig. 4C) were also observed in high-risk patients. There were also higher DMFS, PFS, and OS rates in the low-risk groups observed in the validation set (Fig. 4D, E, and F). The number of events and 5-year DMFS, PFS, and OS rates for the relevant groups are listed in Supplementary Tables 7 and 8. The baseline characteristics (with age, sex, cancer stages, EBV DNA, and treatment regimens included) of patients in the high-risk and low-risk groups in predicting DMFS, PFS, and OS are presented in Supplementary Tables 9, 10 and 11, respectively.

### Potential role of lipid alterations in NPC prognosis

**Fig. 5:**
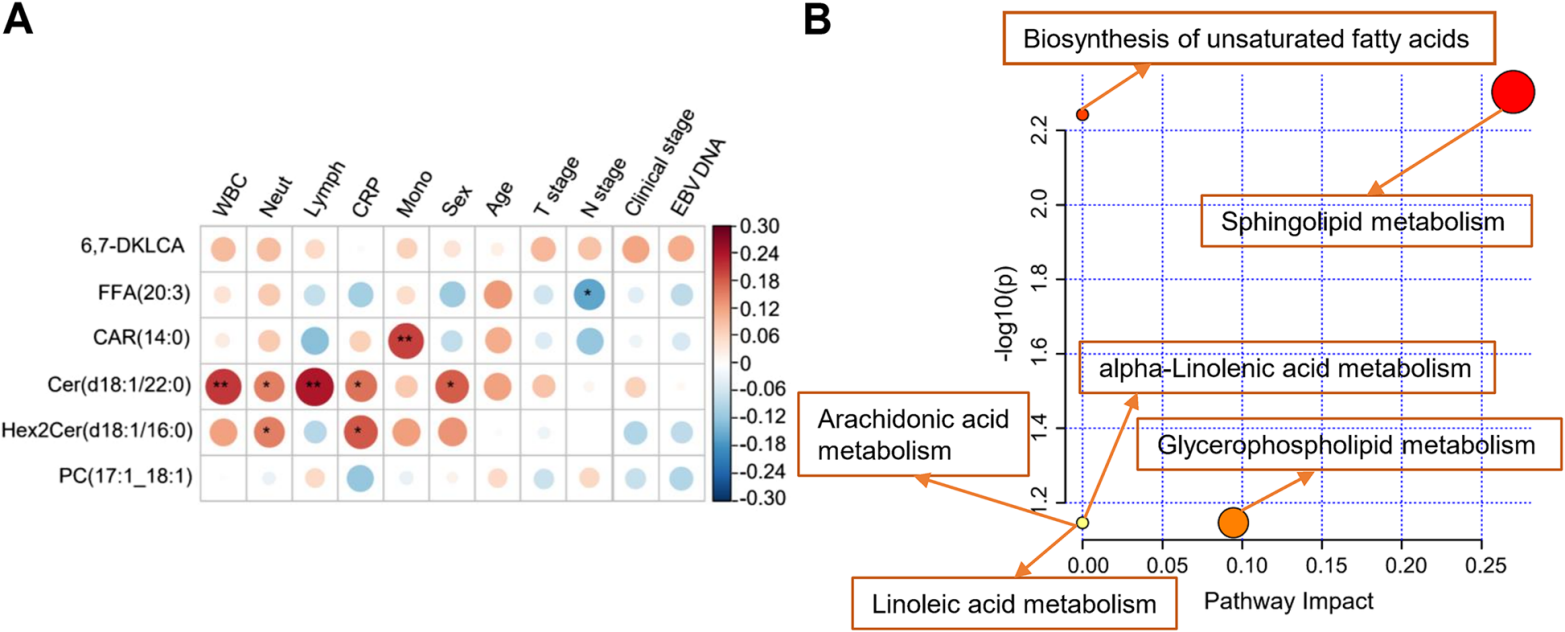
Potential role of lipid alterations in NPC prognosis. **A** Spearman correlation of the 6 plasma lipids with clinical indices. The color of the circle represents the correlation value. Red represents the positive correlation and blue represents negative. The stars within the circle represent the significance of the correlation (*: *P* value < 0.05; **: *P* value < 0.01). **B** Enriched pathways of the 6 predictive lipids by KEGG pathway analysis. Abbreviations: FFA, free fatty acid; CAR, acylcarnitine; Cer, ceramides; Hex2Cer, dihexosylceramide; PC, phosphatidylcholines; DKLCA, diketolithocholic acid; WBC, white blood cell count; Neut, neutrophil count; Lymph, lymphocyte count; CRP, C-reactive protein; Mono, monocyte count; EBV, Epstein-Barr virus

Immunity is considered to be pivotal in in cancer development and treatment. Lipid metabolism, including cholesterol and FA metabolism, can affect the activity and antitumor immunity of CD8 + T cell [27–29]. The study investigated the correlation between plasma lipid levels and clinical indices to explore the potential effect of lipid alterations on NPC prognosis. As shown in Fig. 5A, the six differentially expressed lipid metabolites (Cer(d18:1/22:0), FFA(20:3), CAR(14:0), 6,7-DKLCA, PC(17:1_18:1), and Hex2Cer (d18:1/16:0)), especially Cer(d18:1/22:0) and Hex2Cer (d18:1/16:0), were significantly positively correlated with immunity- and inflammation-associated biomarkers, with C-reactive protein (CRP) and counts of white blood cell (WBC), neutrophil, lymphocyte and monocyte included. According to the KEGG pathway analysis, the six predictive lipids were mainly enriched in metabolic pathways, including biosynthesis of unsaturated FAs and metabolism of sphingolipid, glycerophospholipid, arachidonic acid, linoleic acid and alpha-linolenic acid (Fig. 5B and Supplementary Table 12). The closer to the upper right corner, the more reliable the metabolic pathway.

## Discussion

The study detected quantitative data of 665 plasma lipid metabolites consisting of 27 lipid classes/subclasses from 179 patients with NPC using the widely targeted lipidomics constructed principally on UPLC-MS/MS. Specifically, Cer(d18:1/22:0), FFA(20:3), CAR(14:0), 6,7-DKLCA, PC(17:1_18:1), and Hex2Cer(d18:1/16:0) were observed strong association with the DMFS. A deep learning survival model based on lipids and clinical biomarkers was developed to accurately predict DMFS in patients with LANPC. The proposed survival model consistently showed satisfying performance across independent data sets. Moreover, promising lipid alterations were suggested to affect survival by disrupting the immune and inflammatory systems and metabolic pathways.

As a broad array of studies have reported that lipid metabolism plays an vital role in the tumor microenvironment and prognostic heterogeneity [14, 15, 30, 31], the study mainly aimed to build a robust survival model based on lipidomics to predict DMFS in patients with LANPC. First, the lipidomic methodology (UPLC-MS/MS) offered a broad coverage of the lipidome, achieving excellent performance in numerous clinical studies [32, 33]. A single internal standard per lipid species was included to obtain exact quantification, contributing to a straightforward and clear overview of altered or unbalanced individual lipidomic patterns. Second, six lipid species (Cer(d18:1/22:0), FFA(20:3), CAR(14:0), 6,7-DKLCA, PC(17:1_18:1), and Hex2Cer(d18:1/16:0)) were recognized as significant distant metastasis-associated lipids. As the quantification of lipids relied on respective internal standards, the proposed model enabled the quantitative detection of fewer lipids, making it possible for clinical applications. Third, compared with the baseline model based only on clinical biomarkers, the proposed deep learning model performed outstandingly in terms of survival prediction and risk stratification. These results suggest that lipid biomarkers provide more detailed and beneficial information in the prediction results, thus yielding promising performance in risk stratification, complementing the TNM stages and other clinical variables.

Cells of cancer and other types in the tumor microenvironment use a variety of means to contend lipids and broadly link their metabolism as part of a malleable metabolic and cell signaling reprogramming. Under acidosis, ferroptosis-mediated anticancer effects were selectively induced by N-3 and n-6 polyunsaturated FAs [34]. Compared with nonmalignant tissues, Marien et al*.* initially observed decreasing SMs and specifically increasing PIs in non-small cell lung cancer [35]. The increase in FFAs activated PI3K/AKT and Wnt/beta-catenin signaling pathway, and it was observed involved in the cellular stemness of cancer [36]. Wang et al. characterized FFA(18:1), PC-O(32:1) and TG(18:1_17:1_18:2) as prognostic factors of hepatocellular carcinoma [37]. The TG profile was significantly associated with postoperative disease-free survival in colorectal cancer [38]. Moreover, Chen et al. reported that elevated circulating TG levels were associated with poorer survival of malignant mesothelioma, whereas increased monohexosylceramide might be beneficial [39]. These studies mentioned above reveal that metabolic reprogramming of lipid exhibited significant prognostic values in cancers. Therefore, to discover prognostic factors and therapeutic targets so as to understand the pathophysiological mechanisms related to lipids, NPC-related metabolism and dysfunctions of lipids are worth in-depth mining.

The study demonstrated several associations of the six significant prognosis-associated lipid species with demographics, cancer stage, immune and inflammatory systems, and metabolic pathways (Fig. 5 and Supplementary Table 9). Although not all the findings of the study aligned perfectly, they were generally internally consistent. There were correlations between the six differentially expressed lipid metabolites and clinical indices in the direction of their effect on immune and inflammatory systems and those associated with survival. For example, Hex2Cer (d18:1/16:0) (HR, 0.53; 95% CI, 0.33–0.85; *P* = 0.009) was positively correlated with CRP and counts of WBC, neutrophil and lymphocyte. Tremendous attention has been paid to ceramides as a tumor suppressor in combination therapy for suppressors [40, 41]. Ceramide dysregulation has been shown to spread and manipulate cell signaling events by regulating exosome lipid rafts [42]. Exosome reprogramming allows the growth of metastases and the escape of immune surveillance [43–45]. Moreover, these six lipids were mainly enriched in the sphingolipid metabolic pathway. Sphingolipid is known as lipid signaling molecules regulating inflammation and suppressing tissue damage [46, 47]. Piazzesi et al*.* determined that sphingolipids showed bipolarity in the development and progression of different cancers, either helping or hindering the transformation of healthy cells to cancerous ones [48]. Although not entirely consistent with other studies, altered or unbalanced lipid patterns observed by the study may affect NPC prognosis by interfering with sphingolipid metabolism and the immune and inflammatory systems, indicating the potentially new intervention strategies for modifying sphingolipid metabolism and attenuating disease progression.

### Comparisons with other studies and what does the current work add to the existing knowledge

Although dysregulated lipid metabolism is reported strongly associated with cancer progression, the prognostic value of lipid alterations in NPC remained unclear. Compared with other studies, the study measured the quantification of lipids by absolute analysis method, with considerable insight into the altered lipid metabolism. Moreover, the method of neural network model establishment called DeepSurv enabled the stable prediction performance with preferable fitting effect, ensuring that the screened lipids had reliable predictive value.

### Study strengths and limitations

The study successfully revealed six plasma lipid predictors for the survival of patients with LANPC. By widely targeted quantitative lipidomics, this study measured and quantified 655 lipids, providing insights into the distribution of plasma lipid profiling in patients with LANPC. By deep learning prognostic model, this study revealed plasma lipid predictors for NPC, indicating the targets of intervention to attenuate disease progression.

There are also some main limitations. One main challenge lies in the fact that identifying and quantifying lipid metabolites by plasma lipidomic analyses might be a physical and financial burden to patients, which is detrimental to the use of the proposed model in a clinical setting. Moreover, although the study screened out six prognosis-associated lipid species and tentatively explored their potential roles, the underlying mechanism remains to be elucidated. Finally, regarding to the limited sample size, the findings from these populations need to be validated in future studies with larger independent cohorts.

## Conclusions

Widely targeted quantitative lipidomics reveals plasma lipid predictors and pathway dysregulation for LANPC. The proposed prognostic model based on widely targeted quantitative lipidomics demonstrated superior performance in predicting metastasis in patients with LANPC. Monitoring of these lipid biomarkers during treatment of NPC can understand the heterogeneous prognosis of NPC. In addition, the subsequent intervention by the clinician can mitigate disease progression.

## Supplementary Information

**Additional file 1:** **Supplementary Figure 1.** Identification and distribution of lipid components. (A) Numbers of lipid species in lipid classes/subclasses detected by lipidomics analysis. Circular diagram of lipid subclass composition in non-metastatic group (B) and metastatic group (C). **Supplementary Figure 2.** The overlapped total ion chromatogram (TIC) of the plasma mixtures in negative and positive modes. **Supplementary Table 1.** Information of the instrument used in lipidomics analyses. **Supplementary Table 2.** Information of the reagents and internal standards used in lipidomics analyses. **Supplementary Table 3.** 655 unique lipids were identified and quantified. **Supplementary Table 4.** Baseline characteristics of patients in the training set and validation set. **Supplementary Table 5.** Associations of lipid species with distant metastasis-free survival (DMFS) of Nasopharyngeal carcinoma (NPC) by univariate Cox regression analysis. Only the lipid species with *P* value<0.05 was demonstrated in the table. **Supplementary Table 6.** C-index of the models based on clinical biomarkers with / without lipid biomarkers in predicting DMFS, PFS, and OS. **Supplementary Table 7.** Number of events in the high-risk and low-risk groups. **Supplementary Table 8.** 5-year DMFS, PFS, and OS estimates for the high-risk and low-risk groups. **Supplementary Table 9.** Baseline characteristics of patients in the high-risk and low-risk groups in predicting DMFS. **Supplementary Table 10.** Baseline characteristics of patients in the high-risk and low-risk groups in predicting PFS. **Supplementary Table 11.** Baseline characteristics of patients in the high-risk and low-risk groups in predicting OS. **Supplementary Table 12.** Pathway enrichment and topology analysis result of the predictive lipid species.

## Data Availability

The datasets generated and/or analyzed during the current study are available in the Research Data Deposit platform (RDDA2022448584), and can be inquired at www.researchdata.org.cn. The data supporting the findings of this study are available from the corresponding authors upon reasonable request and this option would be available upon publication.

## Abbreviations

NPC: Nasopharyngeal carcinoma
LANPC: Locoregionally advanced NPC
DMFS: Distant metastasis-free survival
PFS: Progression-free survival
OS: Overall survival
BMI: Body mass index
T: Tumor
N: Lymph node
CHO: Cholesterol
TG: Triglyceride
HDL: High-density lipoprotein cholesterol
LDL: Low-density lipoprotein cholesterol
WBC: White blood cell
CRP: C-reactive protein
LDH: Lactate dehydrogenase
EBV: Epstein-Barr virus
CCRT: Concurrent chemoradiotherapy
ICT: Induction chemotherapy
HR: Hazard ratio
C-index: Concordance index
ROC: Receiver operating characteristic
AUC: Area under the ROC curve
CI: Confidence interval
BA: Bile acid
FFA: Free fatty acid
LPG: Lysophosphatidylglycerol
LPI: Lysophosphatidylinositol
LPS: Lysophosphatidylserine
PI: Phosphatidylinositol
CerP: Ceramide-1-phosphate
CAR: Acylcarnitine
Cho: Cholesterol
CE: Cholesteryl ester
SPH: Sphingosine
Cer: Ceramide
CoQ: Coenzyme Q
DG: Diacylglycerol
HerCer: Hexosylceramide
LPC: Lysophophatidylcholine
LPC-O: Alkyl-lysophophatidylcholine
LPE: Lysophosphatidylethanolamine
LPE-P: Alkenyl-lysophosphatidylethanolamine
PC: Phosphatidylcholine
PC-O: Alkylglycerophosphocholine
PE: Phosphatidylethanolamine
PE-P: Alkenylglycerophosphoethanolamine
PS: Phosphatidylserine
SM: Sphingomyelin
TG: Triacylglycerol

## References

[1] Chen YP, Chan ATC, Le QT, Blanchard P, Sun Y, Ma J. Nasopharyngeal carcinoma. Lancet. 2019; 10.1016/S0140-6736(19)30956-0. 10.1016/S0140-6736(19)30956-0 31178151

[2] Bray F, Ferlay J, Soerjomataram I, Siegel RL, Torre LA, Jemal A. Global cancer statistics 2018: GLOBOCAN estimates of incidence and mortality worldwide for 36 cancers in 185 countries. CA Cancer J Clin. 2018; 10.3322/caac.21492. 10.3322/caac.21492 30207593

[3] Tang LL, Chen WQ, Xue WQ, He YQ, Zheng RS, Zeng YX, et al. Global trends in incidence and mortality of nasopharyngeal carcinoma. Cancer Lett. 2016; 10.1016/j.canlet.2016.01.040. 10.1016/j.canlet.2016.01.040 26828135

[4] Mao YP, Xie Fy Fau - Liu L-Z, Liu Lz Fau - Sun Y, Sun Y Fau - Li L, Li L Fau - Tang L-L, Tang Ll Fau - Liao X-B, et al. Re-evaluation of 6th edition of AJCC staging system for nasopharyngeal carcinoma and proposed improvement based on magnetic resonance imaging. Int J Radiat Oncol Biol Phys. 2009 10.1016/j.ijrobp.2008.07.062. 10.1016/j.ijrobp.2008.07.062 19153016

[5] Lan XW, Xiao Y, Zou XB, Zhang XM, OuYang PY, Xie FY. Outcomes of adding induction chemotherapy to concurrent chemoradiotherapy for stage T3N0–1 nasopharyngeal carcinoma: a propensity-matched study. Onco Targets Ther. 2017; 10.2147/OTT.S133917. eCollection 2017. 10.2147/OTT.S133917 PMC5546817 28814884

[6] Wolrab D, Jirasko R, Cifkova E, Horing M, Mei D, Chocholouskova M, et al. Lipidomic profiling of human serum enables detection of pancreatic cancer. Nat Commun. 2022; 10.1038/s41467-021-27765-9. 10.1038/s41467-021-27765-9 PMC8748654 35013261

[7] Lin HM, Mahon KL, Weir JM, Mundra PA, Spielman C, Briscoe K, et al. A distinct plasma lipid signature associated with poor prognosis in castration-resistant prostate cancer. Int J Cancer. 2017; 10.1002/ijc.30903. 10.1002/ijc.30903 28741687

[8] Balaban S, Nassar ZD, Zhang AY, Hosseini-Beheshti E, Centenera MM, Schreuder M, et al. Extracellular Fatty Acids Are the Major Contributor to Lipid Synthesis in Prostate Cancer. Mol Cancer Res. 2019; 10.1158/1541-7786.MCR-18-0347. 10.1158/1541-7786.MCR-18-0347 30647103

[9] Butler LM, Perone Y, Dehairs J, Lupien LE, de Laat V, Talebi A, et al. Lipids and cancer: Emerging roles in pathogenesis, diagnosis and therapeutic intervention. Adv Drug Deliv Rev. 2020; 10.1016/j.addr.2020.07.013. 10.1016/j.addr.2020.07.013 PMC7736102 32711004

[10] Cirri P, Chiarugi P. Cancer associated fibroblasts: the dark side of the coin. Am J Cancer Res. 2011; not available. Available from: https://www.ncbi.nlm.nih.gov/pmc/articles/PMC3186047/. PMC3186047 21984967

[11] Nieman KM, Romero IL, Van Houten B, Lengyel E. Adipose tissue and adipocytes support tumorigenesis and metastasis. Biochim Biophys Acta. 2013; 10.1016/j.bbalip.2013.02.010. 10.1016/j.bbalip.2013.02.010 PMC3742583 23500888

[12] Wang X, Zeng C, Lin J, Chen T, Zhao T, Jia Z, et al. Metabonomics approach to assessing the modulatory effects of St John's wort, ginsenosides, and clomipramine in experimental depression. J Proteome Res. 2012; 10.1021/pr300891v. 10.1021/pr300891v 23110693

[13] Guo Y, Wang X, Qiu L, Qin X, Liu H, Wang Y, et al. Probing gender-specific lipid metabolites and diagnostic biomarkers for lung cancer using Fourier transform ion cyclotron resonance mass spectrometry. Clin Chim Acta. 2012; 10.1016/j.cca.2012.08.010. 10.1016/j.cca.2012.08.010 22906735

[14] Hilvo M, Gade S, Hyotylainen T, Nekljudova V, Seppanen-Laakso T, Sysi-Aho M, et al. Monounsaturated fatty acids in serum triacylglycerols are associated with response to neoadjuvant chemotherapy in breast cancer patients. Int J Cancer. 2014; 10.1002/ijc.28491. 10.1002/ijc.28491 24114462

[15] Lin L, Ding Y, Wang Y, Wang Z, Yin X, Yan G, et al. Functional lipidomics: Palmitic acid impairs hepatocellular carcinoma development by modulating membrane fluidity and glucose metabolism. Hepatology. 2017; 10.1002/hep.29033. 10.1002/hep.29033 28073184

[16] Reichl B, Niederstaetter L, Boegl T, Neuditschko B, Bileck A, Gojo J, et al. Determination of a Tumor-Promoting Microenvironment in Recurrent Medulloblastoma: A Multi-Omics Study of Cerebrospinal Fluid. Cancers (Basel). 2020; 10.3390/cancers12061350. 10.3390/cancers12061350 PMC7352284 32466393

[17] Ni Y, Xie G, Jia W. Metabonomics of human colorectal cancer: new approaches for early diagnosis and biomarker discovery. J Proteome Res. 2014; 10.1021/pr500443c. 10.1021/pr500443c 25105552

[18] Doria ML, Cotrim Z, Macedo B, Simoes C, Domingues P, Helguero L, et al. Lipidomic approach to identify patterns in phospholipid profiles and define class differences in mammary epithelial and breast cancer cells. Breast Cancer Res Treat. 2012; 10.1007/s10549-011-1823-5. 10.1007/s10549-011-1823-5 22037781

[19] Tian Y, Wang Z, Liu X, Duan J, Feng G, Yin Y, et al. Prediction of Chemotherapeutic Efficacy in Non-Small Cell Lung Cancer by Serum Metabolomic Profiling. Clin Cancer Res. 2018; 10.1158/1078-0432.CCR-17-2855. 10.1158/1078-0432.CCR-17-2855 29437793

[20] Chen X, Li Y, Li X, Cao X, Xiang Y, Xia W, et al. An interpretable machine learning prognostic system for locoregionally advanced nasopharyngeal carcinoma based on tumor burden features. Oral Oncol. 2021; 10.1016/j.oraloncology.2021.105335. 10.1016/j.oraloncology.2021.105335 34023742

[21] Katzman JL, Shaham U, Cloninger A, Bates J, Jiang T, Kluger Y. DeepSurv: personalized treatment recommender system using a Cox proportional hazards deep neural network. BMC Med Res Methodol. 2018; 10.1186/s12874-018-0482-1. 10.1186/s12874-018-0482-1 PMC5828433 29482517

[22] Tang LQ, Li CF, Li J, Chen WH, Chen QY, Yuan LX, et al. Establishment and Validation of Prognostic Nomograms for Endemic Nasopharyngeal Carcinoma. J Natl Cancer Inst. 2016; 10.1093/jnci/djv291. 10.1093/jnci/djv291 26467665

[23] Gerds TA, Kattan MW, Schumacher M, Yu C. Estimating a time-dependent concordance index for survival prediction models with covariate dependent censoring. Stat Med. 2013; 10.1002/sim.5681. 10.1002/sim.5681 23172755

[24] Hothorn T, Zeileis A. Partykit: A modular toolkit for recursive partytioning in R. Journal of Machine Learning Research. 2015; not available. Available from: http://apps.webofknowledge.com/full_record.do?product=UA&search_mode=GeneralSearch&qid=1&SID=5Dj4OuJDJuMZOczyHFJ&page=1&doc=1.

[25] Hothorn T, Hornik K, Zeileis AJJoC, statistics G. Unbiased recursive partitioning: A conditional inference framework. 2006; 10.1198/106186006X133933.

[26] Hosmer DW, Lemeshow S, Sturdivant RX. Applied Logistic Regression. 4th ed. New Jersey: John Wiley & Sons, Inc.; 2013. 10.1002/9781118548387.

[27] Ma X, Bi E, Lu Y, Su P, Huang C, Liu L, et al. Cholesterol Induces CD8(+) T Cell Exhaustion in the Tumor Microenvironment. Cell Metab. 2019; 10.1016/j.cmet.2019.04.002. 10.1016/j.cmet.2019.04.002 PMC7061417 31031094

[28] Yang W, Bai Y, Xiong Y, Zhang J, Chen S, Zheng X, et al. Potentiating the antitumour response of CD8(+) T cells by modulating cholesterol metabolism. Nature. 2016; 10.1038/nature17412. 10.1038/nature17412 PMC4851431 26982734

[29] Zhang Y, Kurupati R, Liu L, Zhou XY, Zhang G, Hudaihed A, et al. Enhancing CD8(+) T Cell Fatty Acid Catabolism within a Metabolically Challenging Tumor Microenvironment Increases the Efficacy of Melanoma Immunotherapy. Cancer Cell. 2017; 10.1016/j.ccell.2017.08.004. 10.1016/j.ccell.2017.08.004 PMC5751418 28898698

[30] Liao P, Wang W, Wang W, Kryczek I, Li X, Bian Y, et al. CD8(+) T cells and fatty acids orchestrate tumor ferroptosis and immunity via ACSL4. Cancer Cell. 2022; 10.1016/j.ccell.2022.02.003. 10.1016/j.ccell.2022.02.003 PMC9007863 35216678

[31] Lanfear DE, Gibbs JJ, Li J, She R, Petucci C, Culver JA, et al. Targeted Metabolomic Profiling of Plasma and Survival in Heart Failure Patients. JACC Heart Fail. 2017; 10.1016/j.jchf.2017.07.009. 10.1016/j.jchf.2017.07.009 PMC5679305 29096792

[32] Song JW, Lam SM, Fan X, Cao WJ, Wang SY, Tian H, et al. Omics-Driven Systems Interrogation of Metabolic Dysregulation in COVID-19 Pathogenesis. Cell Metab. 2020; 10.1016/j.cmet.2020.06.016. 10.1016/j.cmet.2020.06.016 PMC7311890 32610096

[33] Qin M, Zhu Q, Lai W, Ma Q, Liu C, Chen X, et al. Insights into the prognosis of lipidomic dysregulation for death risk in patients with coronary artery disease. Clin Transl Med. 2020; 10.1002/ctm2.189. 10.1002/ctm2.189 PMC7522592 32997403

[34] Dierge E, Debock E, Guilbaud C, Corbet C, Mignolet E, Mignard L, et al. Peroxidation of n-3 and n-6 polyunsaturated fatty acids in the acidic tumor environment leads to ferroptosis-mediated anticancer effects. Cell Metab. 2021; 10.1016/j.cmet.2021.05.016. 10.1016/j.cmet.2021.05.016 34118189

[35] Marien E, Meister M, Muley T, Fieuws S, Bordel S, Derua R, et al. Non-small cell lung cancer is characterized by dramatic changes in phospholipid profiles. Int J Cancer. 2015; 10.1002/ijc.29517. 10.1002/ijc.29517 PMC4503522 25784292

[36] Liu S, Sun Y, Hou Y, Yang L, Wan X, Qin Y, et al. A novel lncRNA ROPM-mediated lipid metabolism governs breast cancer stem cell properties. J Hematol Oncol. 2021; 10.1186/s13045-021-01194-z. 10.1186/s13045-021-01194-z PMC8555326 34715882

[37] Wang Q, Tan Y, Jiang T, Wang X, Li Q, Li Y, et al. Metabolic Reprogramming and Its Relationship to Survival in Hepatocellular Carcinoma. Cells. 2022; 10.3390/cells11071066. 10.3390/cells11071066 PMC8997969 35406630

[38] Ecker J, Benedetti E, Kindt ASD, Horing M, Perl M, Machmuller AC, et al. The Colorectal Cancer Lipidome: Identification of a Robust Tumor-Specific Lipid Species Signature. Gastroenterology. 2021; 10.1053/j.gastro.2021.05.009. 10.1053/j.gastro.2021.05.009 34000281

[39] Chen Z, Song S, Yang C, Dai Z, Gao Y, Li N, et al. Lipid profiling in malignant mesothelioma reveals promising signatures for diagnosis and prognosis: A plasma-based LC-MS lipidomics study. Clin Chim Acta. 2022; 10.1016/j.cca.2021.11.024. 10.1016/j.cca.2021.11.024 34843704

[40] Moro K, Nagahashi M, Gabriel E, Takabe K, Wakai T. Clinical application of ceramide in cancer treatment. Breast Cancer. 2019; 10.1007/s12282-019-00953-8. 10.1007/s12282-019-00953-8 PMC7315770 30963461

[41] Jeffries KA, Krupenko NI. Ceramide Signaling and p53 Pathways. Adv Cancer Res. 2018; 10.1016/bs.acr.2018.04.011. 10.1016/bs.acr.2018.04.011 PMC6361168 30060809

[42] Elsherbini A, Bieberich E. Ceramide and Exosomes: A Novel Target in Cancer Biology and Therapy. Adv Cancer Res. 2018; 10.1016/bs.acr.2018.05.004. 10.1016/bs.acr.2018.05.004 PMC6109973 30060807

[43] Chiarugi P, Cirri P. Metabolic exchanges within tumor microenvironment. Cancer Lett. 2016; 10.1016/j.canlet.2015.10.027. 10.1016/j.canlet.2015.10.027 26546872

[44] Dorsam B, Reiners KS, von Strandmann EP. Cancer-derived extracellular vesicles: friend and foe of tumour immunosurveillance. Philos Trans R Soc Lond B Biol Sci. 2018; 10.1098/rstb.2016.0481. 10.1098/rstb.2016.0481 PMC5717436 29158311

[45] Ruivo CF, Adem B, Silva M, Melo SA. The Biology of Cancer Exosomes: Insights and New Perspectives. Cancer Res. 2017; 10.1158/0008-5472.CAN-17-0994. 10.1158/0008-5472.CAN-17-0994 29162616

[46] Nixon GF. Sphingolipids in inflammation: pathological implications and potential therapeutic targets. Br J Pharmacol. 2009; 10.1111/j.1476-5381.2009.00281.x. 10.1111/j.1476-5381.2009.00281.x PMC2785521 19563535

[47] Cyr A, Zhong Y, Reis SE, Namas RA, Amoscato A, Zuckerbraun B, et al. Analysis of the Plasma Metabolome after Trauma, Novel Circulating Sphingolipid Signatures, and In-Hospital Outcomes. J Am Coll Surg. 2021; 10.1016/j.jamcollsurg.2020.12.022. 10.1016/j.jamcollsurg.2020.12.022 PMC11875205 33453380

[48] Piazzesi A, Afsar SY, van Echten-Deckert G. Sphingolipid metabolism in the development and progression of cancer: one cancer's help is another's hindrance. Mol Oncol. 2021; 10.1002/1878-0261.13063. 10.1002/1878-0261.13063 PMC8637577 34289244

