# Supplementary material for: Widely targeted quantitative lipidomics and prognostic model reveal plasma lipid predictors for nasopharyngeal carcinoma
Source: Lipids Health Dis. 2023 Jun 26;22:81. doi: 10.1186/s12944-023-01830-2 (PMC10294458; doi:10.1186/s12944-023-01830-2)
Supplement: Supplementary file 1 — Additional file 1: Supplementary Figure 1. Identification and distribution of lipid components. (A) Numbers of lipid species in lipid classes/subclasses detected by lipidomics analysis. Circular diagram of lipid subclass composition in non-metastatic group (B) and metastatic group (C). Supplementary Figure 2. The overlapped total ion chromatogram (TIC) of the plasma mixtures in negative and positive modes. Supplementary Table 1. Information of the instrument used in lipidomics analyses. Supplementary Table 2. Information of the reagents and internal standards used in lipidomics analyses. Supplementary Table 3. 655 unique lipids were identified and quantified. Supplementary Table 4. Baseline characteristics of patients in the training set and validation set. Supplementary Table 5. Associations of lipid species with distant metastasis-free survival (DMFS) of Nasopharyngeal carcinoma (NPC) by univariate Cox regression analysis. Only the lipid species with P value<0.05 was demonstrated in the table. Supplementary Table 6. C-index of the models based on clinical biomarkers with / without lipid biomarkers in predicting DMFS, PFS, and OS. Supplementary Table 7. Number of events in the high-risk and low-risk groups. Supplementary Table 8. 5-year DMFS, PFS, and OS estimates for the high-risk and low-risk groups. Supplementary Table 9. Baseline characteristics of patients in the high-risk and low-risk groups in predicting DMFS. Supplementary Table 10. Baseline characteristics of patients in the high-risk and low-risk groups in predicting PFS. Supplementary Table 11. Baseline characteristics of patients in the high-risk and low-risk groups in predicting OS. Supplementary Table 12. Pathway enrichment and topology analysis result of the predictive lipid species. [file 12944_2023_1830_MOESM1_ESM.docx]

Widely targeted quantitative lipidomics and prognostic model reveal plasma lipid predictors for nasopharyngeal carcinoma

*SUPPLEMENTARY DATA*

*Supplementary Materials*

*Supplementary Figure 1.*

*Supplementary Figure 2.*

*Supplementary Table 1.*

*Supplementary Table 2.*

*Supplementary Table 3.*

*Supplementary Table 4.*

*Supplementary Table 5.*

*Supplementary Table 6.*

*Supplementary Table 7.*

*Supplementary Table 8.*

*Supplementary Table 9.*

*Supplementary Table 10.*

*Supplementary Table 11.*

*Supplementary Table 12.*

SUPPLEMENTARY DATA

**Supplementary Materials**

1. **Method**
   1. *Treatment and follow-up*

All patients received IMRT. The total radiation doses were 66–72 Gy in 30-35 fractions and treatment was delivered once daily, over 5 fractions per week. All patients underwent platinum-based chemotherapy, including concurrent chemoradiotherapy (CCRT) with or without induction chemotherapy (ICT). ICT consisted of cisplatin with 5-fluorouracil, taxanes, or both triweekly for two to three cycles. Concurrent chemotherapy consisting of cisplatin was administered weekly or triweekly during radiotherapy. Patients were followed up every 3 months over the first 2 years after radical therapy, every 6 months over the next three years, and once yearly thereafter. Follow-up included measurements of the plasma Epstein-Barr virus (EBV) DNA concentration, indirect or direct nasopharyngoscopy, plain and contrast-enhanced head and neck MRI, chest X-ray/plain and contrast-enhanced computed tomography (CT), and abdominal sonography/plain and contrast-enhanced CT.

- 1. *Lipidomics Analyses*
     1. *Plasma sample collection*

Fasting for at least 8 h was required before collecting blood samples in the morning. The whole blood sample was collected in EDTA anticoagulant tube and separated into plasma and hemocyte within 2 h with centrifugation at 1000 g about 10 min at 4℃. The plasma was then stored at −80℃ for future analysis.

- - 1. *Extraction and Analysis of Lipid Metabolites*

Plasma samples were taken out from the−80℃ freezer and thawed at room temperature. After thawing, all samples were centrifuged and vortexed for 10 s to ensure uniform mixing, then these samples were centrifuged at 3,000 g for 5 min at 4℃. After centrifugation, 50 μL of plasma was extracted and transferred to the clean and corresponding numbered Eppendorf tube. Subsequently, 1 ml of lipid extraction solution and internal standards mixture were added to the Eppendorf tube and mixed by vortex for 2 min. The mixed samples were emulsified by sonication for 5 min, mixed with 200 uL water, vortexed for 1 min, and then centrifuged at 4℃ 12,000 g for 10 min. The 500 uL supernatant was collected and dried with nitrogen and re-dissolved with 200 uL of mobile phase B. After vortex oscillation for 1 min, the sample was centrifuged at 14,000 g for 15 min at 4℃, and Ultra Performance Liquid Chromatography and Tandem Mass Spectrometry (UPLC-MS/MS) analysis was carried out.

- - 1. *Liquid Chromatography and Mass Spectrometry*

UPLC (ExionLC^TM^ AD, https://sciex.com.cn/) and MS/MS (QTRAP® 6500+, https://sciex.com.cn/) are the main instrument system for data acquisition. The chromatographic columns from Thermo AccucoreTMC30 (2.6 µm, 2.1 mm×100 mm i.d.) were used. The solvent system was as follows: A, acetonitrile/water (60/40, V/V, 0.1% formic acid, 10 mmol/L ammonium formate); B, acetonitrile/isopropanol (10/90, V/V, 0.1% formic acid, 10 mmol/L ammonium formate). Gradient program was t = 0 min: A/B (80:20, V/V); t = 2.0 min: A/B (70:30, V/V); t = 4 min: A/B (40:60, V/V); t = 9 min: A/B (15:85, V/V), t = 14 min: A/B (10:90, V/V); t = 15.5 min: A/B (5:95, V/V); t = 17.3 min: A/B (5:95, V/V); t = 17.5 min: A/B (80:20, V/V); t = 20 min: A/B (80:20, V/V); and flow rate was 0.35 ml/min, with temperature was 45℃. Subsequently, the effluent was alternatively connected to an electrospray ionization (ESI) -triple quadrupole-linear ion trap (QTRAP)-MS. Linear ion trap and triple quadrupole (QQQ) scans were acquired on a triple quadrupole-linear ion trap mass spectrometer (QTRAP), QTRAP® 6500+ LC-MS/MS System, equipped with an ESI Turbo Ion-Spray interface, operating in positive and negative ion mode and controlled by Analyst 1.6.3 software (Sciex). Lipids contents were detected by MetWare (http://www.metware.cn/) based on the AB Sciex QTRAP 6500 LC-MS/MS platform.

- - 1. *Analysis of quality control*

The calibration and quality control (QC) samples were prepared with the mixed plasma of subjects prior to sample analysis. Every 10 samples to be analyzed were separated by one QC sample for the duration of the detection to monitor repeatability during the analysis. The high overlaps of the total ion flow between different QC samples, that is, the retention time and peak strength are consistent, indicates that the signal stability of the mass spectrum is good at different times.

- - 1. *Qualitative and Quantitative Analysis of lipidomic data*

The Metware database was constructed based on the standard materials to qualitatively analyze the data detected by mass spectrometry. The multiple reaction monitoring (MRM) mode of triple quadrupole mass spectrometry was applied for the quantification of analytes.

- - - 1. *Qualitative Analysis*

Through collecting ion flow intensity (Intensity, cps) and retention time (Time, min) of ion detection, the mass spectrometry peaks detected in different samples for each lipid metabolites can be obtained. The characteristic ions of each lipid metabolites were processed by the multiple reaction monitoring based on the Metware database, and then MultiQuant software was performed to analyze the chromatogram review and peak area integration of the off-board mass spectrometry file of the sample. The lipid metabolite structural analysis mainly referred to MassBank, KNAPSAcK, HMDB, Lipidmaps, and METLIN database.

- - - 1. *Quantitative Analysis*

Quantitative analysis was performed using MRM by triple quadrupole mass spectrometry. In MRM mode, the quadrupole firstly screened the precursor ions of the target substance, and excluded the ions of other molecular weight to eliminate the interference initially. After ionization induced by the impact chamber, the precursor ions break and form many fragments, and then a characteristic fragment ion is selected through the triple quadrupole filtration to exclude the interference of non-target ions, accompanying the more accurate quantification and better repeatability. After obtaining the mass spectrometry data of different samples, the chromatographic peaks of all targets were integrated and quantitatively analyzed by internal standard method.

- - - - 1. *The* *calibration curves constructed from* *internal standards*

Prepared 0.0002 nmol/mL, 0.0005 nmol/mL, 0.001 nmol/mL, 0.002 nmol/mL, 0.005 nmol/mL, 0.01 nmol/mL, 0.02 nmol/mL, 0.05 nmol/mL, 0.1 nmol /mL, 0.2 nmol/mL, 0.5 nmol/mL, 1 nmol/mL, 2 nmol/mL, 5 nmol/mL, 10 nmol /mL solutions of different standard compounds, and collected the mass spectrum peak intensity data of the corresponding quantitative signal of standard substance with different concentrations. The calibration curves of different standards were plotted with the Concentration Ratio of external standard and internal standard as abscissa and the Area Ratio of external standard and internal standard as ordinate.

- - - - 1. *The absolute* *concentrations of lipid species*

The ratio of integral peak area of detected samples was substituted into the linear equation of the calibration curves of internal standards for calculation, and further substituted into the following formula to obtain the absolute concentrations of lipid species.

**X=0.001*c*V*v_1_/v_2_/m**

X: the absolute concentration of lipid (nmol/mL);

c: the concentration value obtained by integrating the ratio of peak area into the corresponding calibration curve of internal standard (nmol/mL);

V: the volume of the redissolved solution (μL);

v_1_: the volume of the sample extract (μL);

v_2_: the volume of the supernatant collected (μL);

m: the volume of the plasma sample (μL, the volume of the plasma samples we used in our study is 50 μL).

**
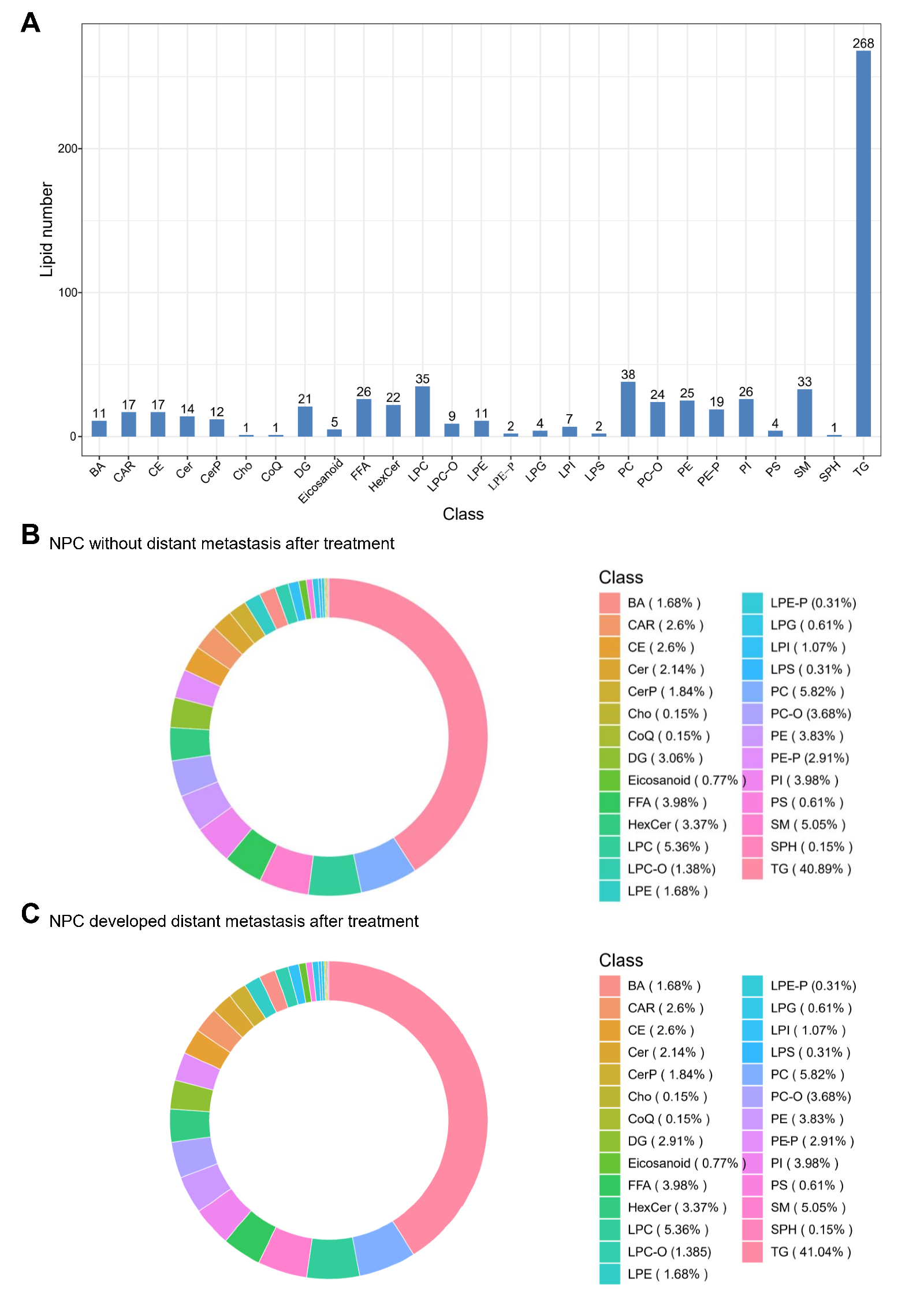
**

**Supplementary Figure 1.** Identification and distribution of lipid components. (A) Numbers of lipid species in lipid classes/subclasses detected by lipidomics analysis. Circular diagram of lipid subclass composition in non-metastatic group (B) and metastatic group (C).

**
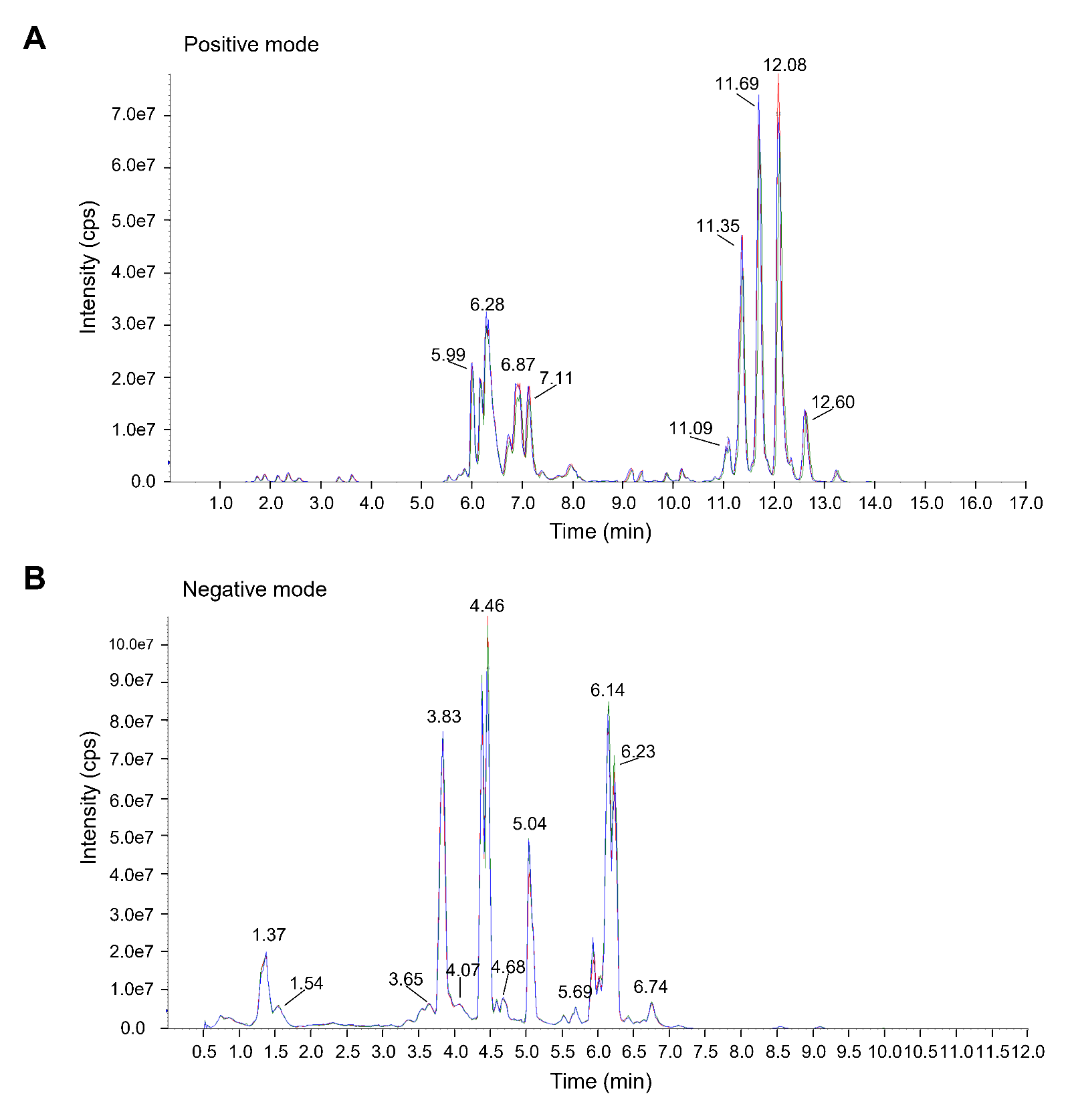
**

**Supplementary Figure 2.** The overlapped total ion chromatogram (TIC) of the plasma mixtures in negative and positive modes.

**Supplementary Table 1. Information of the instrument used in lipidomics analyses.**

| **Instrument** | **Product type** | **Brand** |
| --- | --- | --- |
| LC-MS/MS | QTRAP 6500+ | SCIEX |
| Centrifuge | 5424R | Eppendorf |
| Electronic Balance | AS 60/220.R2 | RADWAG |
| Ball mill | MM400 | Retsch |
| Centrifugal concentrator | CentriVap | LABCONCO |
| Multi-tube vortexer | MIX-200 | Shanghai Jingxin |
| Ultrasonic cleaner | KQ5200E | Kunshan Supmile |

**Supplementary Table 2. Information of the reagents and internal standards used in lipidomics analyses.**

| **Regents** | **Class** | **Brand** |
| --- | --- | --- |
| Methanol | HPLC | Merck |
| Acetonitrile | HPLC | Merck |
| Formic acid | HPLC | Sigma-Aldrich |
| Ammonium formate | HPLC | Fisher |
| Chloroform | HPLC | Fisher |
| Isopropanol | HPLC | Merck |
| Methyl tertiary butyl ether | HPLC | CNW |
| Internal standards* | >99% | Avanti/zzstandard |
| * Internal standards were prepared with a mixture of methylene chloride/methanol and stored at -20°C. | | |

**Supplementary Table 3. 655 unique lipids were identified and quantified.**

| **Lipid subclass** | **Lipid specie** | **Concentration (nmol/mL)** | |
| --- | --- | --- | --- |
|  |  | **mean** | **se** |
| BA | TLCA-3S | 1.117 | 0.495 |
| BA | CDCA | 6.537 | 1.076 |
| BA | UDCA | 4.124 | 4.551 |
| BA | 6,7-DKLCA | 2.883 | 0.793 |
| BA | UCA | 7.632 | 1.928 |
| BA | GCDCA | 2.370 | 2.994 |
| BA | GUDCA | 1.896 | 2.369 |
| BA | LCA-3S | 0.646 | 0.386 |
| BA | GCA | 7.353 | 11.240 |
| BA | TCDCA | 17.034 | 31.806 |
| BA | TCA | 0.672 | 0.297 |
| Eicosanoid | 15-oxoETE | 0.250 | 0.057 |
| Eicosanoid | PGE2 | 0.845 | 2.283 |
| Eicosanoid | 5-iso PGF2VI | 0.039 | 0.012 |
| Eicosanoid | TxB3 | 0.189 | 0.171 |
| Eicosanoid | TXB2 | 0.059 | 0.035 |
| FFA | FFA(12:0) | 0.286 | 0.083 |
| FFA | FFA(14:0) | 1.372 | 0.782 |
| FFA | FFA(15:0) | 0.519 | 0.173 |
| FFA | FFA(16:0) | 60.540 | 23.202 |
| FFA | FFA(17:0) | 0.714 | 0.239 |
| FFA | FFA(18:0) | 34.252 | 9.916 |
| FFA | FFA(20:0) | 1.270 | 0.273 |
| FFA | FFA(22:0) | 2.751 | 0.429 |
| FFA | FFA(24:0) | 0.892 | 0.178 |
| FFA | FFA(26:0) | 0.791 | 0.163 |
| FFA | FFA(16:1) | 5.513 | 5.653 |
| FFA | FFA(17:1) | 0.457 | 0.313 |
| FFA | FFA(18:1) | 70.376 | 52.413 |
| FFA | FFA(20:1) | 2.488 | 1.766 |
| FFA | FFA(22:1) | 0.906 | 0.393 |
| FFA | FFA(24:1) | 0.273 | 0.126 |
| FFA | FFA(18:2) | 72.554 | 54.773 |
| FFA | FFA(20:2) | 1.903 | 1.140 |
| FFA | FFA(22:2) | 0.300 | 0.222 |
| FFA | FFA(18:3) | 1.112 | 0.934 |
| FFA | FFA(20:3) | 0.449 | 0.220 |
| FFA | FFA(22:3) | 0.233 | 0.047 |
| FFA | FFA(20:4) | 4.158 | 2.362 |
| FFA | FFA(22:4) | 0.605 | 0.371 |
| FFA | FFA(20:5) | 0.398 | 0.319 |
| FFA | FFA(22:5) | 2.363 | 1.916 |
| LPG | LPG(16:0) | 0.037 | 0.007 |
| LPG | LPG(18:0) | 0.021 | 0.005 |
| LPG | LPG(18:1) | 0.259 | 0.042 |
| LPG | LPG(18:2) | 0.035 | 0.009 |
| LPI | LPI(16:0) | 1.709 | 1.992 |
| LPI | LPI(18:0) | 29.393 | 8.938 |
| LPI | LPI(16:1) | 0.087 | 0.334 |
| LPI | LPI(18:1) | 21.627 | 10.415 |
| LPI | LPI(18:2) | 27.485 | 13.487 |
| LPI | LPI(20:3) | 3.390 | 3.171 |
| LPI | LPI(20:4) | 21.539 | 9.686 |
| LPS | LPS(18:0) | 1.279 | 2.618 |
| LPS | LPS(20:0) | 0.204 | 0.335 |
| PI | PI(34:0) | 0.373 | 0.115 |
| PI | PI(36:0) | 0.268 | 0.069 |
| PI | PI(32:1) | 0.214 | 0.107 |
| PI | PI(34:1) | 3.162 | 1.316 |
| PI | PI(35:1) | 0.136 | 0.017 |
| PI | PI(36:1) | 1.706 | 0.675 |
| PI | PI(38:1) | 0.107 | 0.003 |
| PI | PI(34:2) | 1.602 | 0.718 |
| PI | PI(35:2) | 0.295 | 0.086 |
| PI | PI(36:2) | 29.912 | 9.273 |
| PI | PI(37:2) | 0.122 | 0.010 |
| PI | PI(38:2) | 0.253 | 0.073 |
| PI | PI(36:3) | 0.210 | 0.103 |
| PI | PI(37:3) | 0.138 | 0.026 |
| PI | PI(38:3) | 1.452 | 0.673 |
| PI | PI(40:3) | 0.105 | 0.008 |
| PI | PI(36:4) | 1.417 | 0.675 |
| PI | PI(37:4) | 0.342 | 0.102 |
| PI | PI(38:4) | 52.222 | 11.099 |
| PI | PI(40:4) | 0.323 | 0.093 |
| PI | PI(38:5) | 0.567 | 0.204 |
| PI | PI(39:5) | 0.120 | 0.020 |
| PI | PI(40:5) | 0.849 | 0.314 |
| PI | PI(38:6) | 0.311 | 0.119 |
| PI | PI(40:6) | 1.499 | 0.631 |
| PI | PI(42:10) | 0.207 | 0.034 |
| CerP | CerP(d18:1/14:0) | 0.525 | 0.778 |
| CerP | CerP(d18:1/16:0) | 15.336 | 23.889 |
| CerP | CerP(d18:1/18:0) | 1.024 | 1.672 |
| CerP | CerP(d18:1/20:0) | 0.638 | 0.888 |
| CerP | CerP(d18:1/24:0) | 2.497 | 3.627 |
| CerP | CerP(d18:1/16:1) | 2.976 | 5.800 |
| CerP | CerP(d18:1/18:1) | 335.588 | 462.982 |
| CerP | CerP(d18:1/24:1) | 14.447 | 25.678 |
| CerP | CerP(d18:1/18:2) | 14.023 | 31.049 |
| CerP | CerP(d18:1/18:3) | 0.439 | 0.672 |
| CerP | CerP(d18:1/20:3) | 1.982 | 2.332 |
| CerP | CerP(d18:1/20:4) | 1.314 | 1.930 |
| CAR | CAR(10:0) | 0.395 | 0.408 |
| CAR | CAR(12:0) | 0.136 | 0.046 |
| CAR | CAR(13:0) | 0.139 | 0.056 |
| CAR | CAR12-OH | 0.092 | 0.009 |
| CAR | CAR(14:0) | 0.111 | 0.016 |
| CAR | CAR(16:0) | 0.366 | 0.094 |
| CAR | CAR(18:0) | 0.206 | 0.040 |
| CAR | CAR(10:1) | 0.317 | 0.232 |
| CAR | CAR(12:1) | 0.192 | 0.097 |
| CAR | CAR(12:1-OH) | 0.090 | 0.004 |
| CAR | CAR(14:1) | 0.188 | 0.104 |
| CAR | CAR(14:1-OH) | 0.095 | 0.008 |
| CAR | CAR(16:1) | 0.168 | 0.054 |
| CAR | CAR(18:1) | 0.452 | 0.144 |
| CAR | CAR(22:1) | 0.108 | 0.008 |
| CAR | CAR(14:2) | 0.139 | 0.058 |
| CAR | CAR(18:2) | 0.380 | 0.137 |
| Cho | Cholesterol | 891.017 | 1120.915 |
| CE | CE(15:0) | 3.490 | 1.097 |
| CE | CE(16:0) | 174.656 | 35.902 |
| CE | CE(17:0) | 2.630 | 0.663 |
| CE | CE(18:0) | 24.492 | 10.783 |
| CE | CE(16:1) | 45.552 | 24.146 |
| CE | CE(17:1) | 7.386 | 3.330 |
| CE | CE(18:1) | 598.525 | 135.246 |
| CE | CE(18:2) | 2361.061 | 485.715 |
| CE | CE(19:2) | 2.966 | 0.780 |
| CE | CE(20:2) | 9.654 | 2.366 |
| CE | CE(18:3) | 98.437 | 47.575 |
| CE | CE(20:3) | 122.831 | 54.150 |
| CE | CE(20:4) | 829.006 | 315.610 |
| CE | CE(22:4) | 8.534 | 2.887 |
| CE | CE(20:5) | 194.971 | 187.534 |
| CE | CE(22:5) | 31.226 | 14.556 |
| CE | CE(22:6) | 301.343 | 165.739 |
| SPH | SPH(d16:1) | 5.063 | 1.394 |
| Cer | Cer(d18:1/16:0) | 0.789 | 0.294 |
| Cer | Cer(d18:1/18:0) | 0.119 | 0.081 |
| Cer | Cer(d18:1/20:0) | 0.155 | 0.073 |
| Cer | Cer(d18:1/22:0) | 2.370 | 0.781 |
| Cer | Cer(d18:1/23:0) | 1.873 | 0.631 |
| Cer | Cer(d18:1/24:0) | 9.069 | 2.966 |
| Cer | Cer(d18:1/25:0) | 0.330 | 0.158 |
| Cer | Cer(d18:1/24:1) | 2.904 | 1.046 |
| Cer | Cer(d16:1/24:0) | 0.174 | 0.108 |
| Cer | Cer(d18:2/18:0) | 0.110 | 0.071 |
| Cer | Cer(d18:2/22:0) | 0.485 | 0.219 |
| Cer | Cer(d18:2/23:0) | 0.513 | 0.235 |
| Cer | Cer(d18:2/24:0) | 1.449 | 0.587 |
| Cer | Cer(d18:2/24:1) | 0.332 | 0.139 |
| CoQ | Coenzyme Q10 | 0.022 | 0.159 |
| DG | DG(16:0_16:0) | 7.687 | 1.744 |
| DG | DG(16:0_18:0) | 31.752 | 5.603 |
| DG | DG(18:0_18:0) | 23.612 | 4.189 |
| DG | DG(16:0_18:1) | 0.736 | 1.692 |
| DG | DG(18:0_18:1) | 0.020 | 0.153 |
| DG | DG(16:1_18:1) | 0.087 | 0.508 |
| DG | DG(16:0_18:2) | 0.900 | 1.759 |
| DG | DG(18:0_18:2) | 0.002 | 0.022 |
| DG | DG(18:1_18:1) | 5.580 | 4.517 |
| DG | DG(16:1_18:2) | 0.019 | 0.130 |
| DG | DG(18:1_18:2) | 8.673 | 6.649 |
| DG | DG(16:0_20:4) | 0.078 | 0.433 |
| DG | DG(18:2_18:2) | 5.193 | 4.124 |
| DG | DG(18:2_18:3) | 0.577 | 1.521 |
| DG | DG(18:1_20:4) | 0.670 | 1.726 |
| DG | DG(18:2_20:4) | 0.705 | 1.723 |
| DG | DG(18:1_20:5) | 0.048 | 0.407 |
| DG | DG(16:0_22:6) | 0.027 | 0.213 |
| DG | DG(16:1_22:6) | 0.000 | 0.000 |
| DG | DG(18:1_22:6) | 0.890 | 2.320 |
| DG | DG(18:2_22:6) | 0.529 | 1.842 |
| HexCer | HexCer(d16:1/18:0) | 0.040 | 0.010 |
| HexCer | HexCer(d16:1/22:0) | 0.016 | 0.007 |
| HexCer | HexCer(d16:1/24:0) | 0.006 | 0.004 |
| HexCer | HexCer(d18:1/16:0) | 0.333 | 0.115 |
| HexCer | HexCer(d18:1/18:0) | 0.045 | 0.019 |
| HexCer | HexCer(d18:1/20:0) | 0.025 | 0.012 |
| HexCer | HexCer(d18:1/22:0) | 0.403 | 0.156 |
| HexCer | HexCer(d18:1/23:0) | 0.139 | 0.049 |
| HexCer | HexCer(d18:1/24:0) | 0.476 | 0.165 |
| HexCer | HexCer(d18:1/16:1) | 0.130 | 0.041 |
| HexCer | HexCer(d18:1/22:1) | 0.048 | 0.023 |
| HexCer | HexCer(d18:1/24:1) | 0.155 | 0.075 |
| HexCer | HexCer(d18:2/22:0) | 0.065 | 0.025 |
| HexCer | HexCer(d18:2/24:0) | 0.068 | 0.026 |
| HexCer | HexCer(d18:2/24:1) | 0.025 | 0.013 |
| HexCer | Hex2Cer(d16:1/16:0) | 0.012 | 0.007 |
| HexCer | Hex2Cer(d18:1/16:0) | 0.709 | 0.229 |
| HexCer | Hex2Cer(d18:1/18:0) | 0.017 | 0.011 |
| HexCer | Hex2Cer(d18:1/22:0) | 0.015 | 0.008 |
| HexCer | Hex2Cer(d18:1/24:0) | 0.027 | 0.012 |
| HexCer | Hex2Cer(d18:2/16:0) | 0.048 | 0.017 |
| HexCer | Hex3Cer(d18:1/16:0) | 0.039 | 0.018 |
| LPC | LPC(14:0/0:0) | 0.853 | 0.396 |
| LPC | LPC(0:0/15:0) | 0.561 | 0.195 |
| LPC | LPC(15:0/0:0) | 0.566 | 0.200 |
| LPC | LPC(0:0/16:0) | 49.324 | 11.128 |
| LPC | LPC(16:0/0:0) | 52.921 | 11.963 |
| LPC | LPC(0:0/17:0) | 1.298 | 0.453 |
| LPC | LPC(17:0/0:0) | 1.527 | 0.471 |
| LPC | LPC(0:0/18:0) | 43.656 | 12.482 |
| LPC | LPC(18:0/0:0) | 46.928 | 12.748 |
| LPC | LPC(0:0/19:0) | 0.189 | 0.049 |
| LPC | LPC(19:0/0:0) | 0.190 | 0.048 |
| LPC | LPC(0:0/20:0) | 0.286 | 0.088 |
| LPC | LPC(20:0/0:0) | 0.285 | 0.085 |
| LPC | LPC(22:0/0:0) | 0.112 | 0.026 |
| LPC | LPC(24:0/0:0) | 0.224 | 0.076 |
| LPC | LPC(26:0/0:0) | 0.203 | 0.087 |
| LPC | LPC(16:1/0:0) | 3.402 | 1.293 |
| LPC | LPC(17:1/0:0) | 0.289 | 0.103 |
| LPC | LPC(0:0/18:1) | 23.222 | 6.241 |
| LPC | LPC(18:1/0:0) | 29.230 | 7.963 |
| LPC | LPC(19:1/0:0) | 0.136 | 0.032 |
| LPC | LPC(0:0/20:1) | 0.586 | 0.162 |
| LPC | LPC(20:1/0:0) | 0.604 | 0.170 |
| LPC | LPC(0:0/18:2) | 38.970 | 12.480 |
| LPC | LPC(18:2/0:0) | 40.235 | 13.319 |
| LPC | LPC(0:0/20:2) | 0.930 | 0.319 |
| LPC | LPC(20:2/0:0) | 0.985 | 0.321 |
| LPC | LPC(18:3/0:0) | 0.997 | 0.638 |
| LPC | LPC(20:3/0:0) | 3.444 | 1.648 |
| LPC | LPC(20:4/0:0) | 9.174 | 3.127 |
| LPC | LPC(22:4/0:0) | 0.270 | 0.107 |
| LPC | LPC(20:5/0:0) | 1.646 | 1.509 |
| LPC | LPC(0:0/22:5) | 0.318 | 0.111 |
| LPC | LPC(22:5/0:0) | 0.365 | 0.138 |
| LPC | LPC(22:6/0:0) | 1.830 | 0.894 |
| LPC-O | LPC(O-16:0) | 1.686 | 0.485 |
| LPC-O | LPC(O-18:0) | 0.216 | 0.050 |
| LPC-O | LPC(O-20:0) | 0.114 | 0.019 |
| LPC-O | LPC(O-22:0) | 0.164 | 0.039 |
| LPC-O | LPC(O-16:1) | 0.424 | 0.101 |
| LPC-O | LPC(O-18:1) | 1.342 | 0.372 |
| LPC-O | LPC(O-20:1) | 0.084 | 0.012 |
| LPC-O | LPC(O-20:2) | 0.098 | 0.019 |
| LPC-O | LPC(O-22:2) | 0.086 | 0.013 |
| LPE | LPE(0:0/16:0) | 5.015 | 1.633 |
| LPE | LPE(16:0/0:0) | 5.049 | 1.698 |
| LPE | LPE(0:0/18:0) | 7.271 | 2.135 |
| LPE | LPE(18:0/0:0) | 7.242 | 2.161 |
| LPE | LPE(0:0/18:1) | 4.707 | 2.250 |
| LPE | LPE(18:1/0:0) | 4.704 | 2.284 |
| LPE | LPE(0:0/18:2) | 9.484 | 4.267 |
| LPE | LPE(18:2/0:0) | 9.619 | 4.260 |
| LPE | LPE(20:3/0:0) | 0.650 | 0.351 |
| LPE | LPE(20:4/0:0) | 4.427 | 1.413 |
| LPE | LPE(22:6/0:0) | 13.322 | 5.156 |
| LPE-P | LPE(P-16:0) | 0.320 | 0.092 |
| LPE-P | LPE(P-18:0) | 0.358 | 0.098 |
| PC | PC(30:0) | 2.683 | 1.149 |
| PC | PC(31:0) | 0.532 | 0.245 |
| PC | PC(32:0) | 23.608 | 6.083 |
| PC | PC(38:0) | 0.451 | 0.170 |
| PC | PC(30:1) | 0.206 | 0.161 |
| PC | PC(32:1) | 19.853 | 11.695 |
| PC | PC(34:1) | 271.868 | 66.385 |
| PC | PC(35:1) | 1.752 | 0.799 |
| PC | PC(32:2) | 2.272 | 1.034 |
| PC | PC(34:2) | 549.978 | 81.872 |
| PC | PC(35:2) | 5.545 | 1.614 |
| PC | PC(36:2) | 265.602 | 66.985 |
| PC | PC(38:2) | 0.551 | 0.230 |
| PC | PC(32:3) | 0.033 | 0.032 |
| PC | PC(34:3) | 9.749 | 4.682 |
| PC | PC(35:3) | 1.225 | 0.459 |
| PC | PC(36:3) | 192.561 | 50.030 |
| PC | PC(37:3) | 0.727 | 0.415 |
| PC | PC(38:3) | 23.112 | 13.139 |
| PC | PC(34:4) | 0.459 | 0.282 |
| PC | PC(35:4) | 0.670 | 0.319 |
| PC | PC(36:4) | 206.340 | 49.702 |
| PC | PC(37:4) | 1.895 | 0.748 |
| PC | PC(38:4) | 134.781 | 39.271 |
| PC | PC(40:4) | 0.523 | 0.292 |
| PC | PC(35:5) | 0.010 | 0.037 |
| PC | PC(36:5) | 10.612 | 11.794 |
| PC | PC(38:5) | 25.003 | 6.244 |
| PC | PC(40:5) | 5.904 | 2.518 |
| PC | PC(36:6) | 0.180 | 0.140 |
| PC | PC(37:6) | 0.314 | 0.196 |
| PC | PC(38:6) | 93.104 | 33.560 |
| PC | PC(39:6) | 1.808 | 0.904 |
| PC | PC(40:6) | 34.839 | 14.931 |
| PC | PC(38:7) | 0.399 | 0.236 |
| PC | PC(40:7) | 3.415 | 1.134 |
| PC | PC(42:7) | 0.078 | 0.058 |
| PC | PC(40:8) | 0.583 | 0.191 |
| PC-O | PC(O-38:0) | 0.325 | 0.109 |
| PC-O | PC(O-30:1) | 1.717 | 0.489 |
| PC-O | PC(O-32:1) | 3.273 | 0.948 |
| PC-O | PC(O-40:1) | 0.323 | 0.164 |
| PC-O | PC(O-32:2) | 0.138 | 0.067 |
| PC-O | PC(O-34:2) | 9.840 | 4.131 |
| PC-O | PC(O-40:2) | 0.172 | 0.100 |
| PC-O | PC(O-42:2) | 0.043 | 0.039 |
| PC-O | PC(O-34:3) | 0.584 | 0.575 |
| PC-O | PC(O-36:3) | 4.725 | 1.729 |
| PC-O | PC(O-38:3) | 0.990 | 0.336 |
| PC-O | PC(O-34:4) | 0.105 | 0.148 |
| PC-O | PC(O-36:4) | 25.150 | 8.751 |
| PC-O | PC(O-38:4) | 2.006 | 0.895 |
| PC-O | PC(O-44:4) | 0.300 | 0.132 |
| PC-O | PC(O-36:5) | 16.363 | 5.720 |
| PC-O | PC(O-38:5) | 21.595 | 6.271 |
| PC-O | PC(O-40:5) | 0.587 | 0.210 |
| PC-O | PC(O-44:5) | 2.160 | 0.731 |
| PC-O | PC(O-36:6) | 0.749 | 0.775 |
| PC-O | PC(O-38:6) | 8.636 | 3.094 |
| PC-O | PC(O-40:6) | 2.795 | 1.343 |
| PC-O | PC(O-38:7) | 5.192 | 2.133 |
| PC-O | PC(O-40:7) | 3.715 | 1.426 |
| PE | PE(32:0) | 0.192 | 0.006 |
| PE | PE(34:0) | 0.195 | 0.008 |
| PE | PE(32:1) | 0.214 | 0.039 |
| PE | PE(34:1) | 0.675 | 0.329 |
| PE | PE(35:1) | 0.200 | 0.014 |
| PE | PE(36:1) | 0.565 | 0.295 |
| PE | PE(34:2) | 1.591 | 0.829 |
| PE | PE(35:2) | 0.223 | 0.027 |
| PE | PE(36:2) | 3.314 | 1.816 |
| PE | PE(36:3) | 0.993 | 0.526 |
| PE | PE(38:3) | 0.426 | 0.232 |
| PE | PE(36:4) | 1.668 | 0.614 |
| PE | PE(37:4) | 0.221 | 0.019 |
| PE | PE(38:4) | 4.598 | 1.717 |
| PE | PE(40:4) | 0.263 | 0.058 |
| PE | PE(36:5) | 0.228 | 0.051 |
| PE | PE(38:5) | 0.840 | 0.305 |
| PE | PE(40:5) | 0.440 | 0.146 |
| PE | PE(36:6) | 0.183 | 0.020 |
| PE | PE(38:6) | 2.915 | 1.398 |
| PE | PE(39:6) | 0.240 | 0.033 |
| PE | PE(40:6) | 2.016 | 0.978 |
| PE | PE(40:7) | 0.443 | 0.141 |
| PE | PE(42:7) | 0.194 | 0.007 |
| PE | PE(40:8) | 0.204 | 0.009 |
| PE-P | PE(P-34:0) | 2.241 | 0.888 |
| PE-P | PE(P-36:0) | 0.550 | 0.552 |
| PE-P | PE(P-34:1) | 0.505 | 0.205 |
| PE-P | PE(P-36:1) | 0.471 | 0.176 |
| PE-P | PE(P-34:2) | 1.704 | 0.803 |
| PE-P | PE(P-36:2) | 2.181 | 0.944 |
| PE-P | PE(P-36:3) | 22.582 | 11.504 |
| PE-P | PE(P-38:3) | 7.373 | 3.800 |
| PE-P | PE(P-40:3) | 0.638 | 0.283 |
| PE-P | PE(P-36:4) | 9.730 | 4.640 |
| PE-P | PE(P-37:4) | 0.675 | 0.301 |
| PE-P | PE(P-38:4) | 9.096 | 3.902 |
| PE-P | PE(P-36:5) | 0.658 | 0.650 |
| PE-P | PE(P-38:5) | 15.033 | 6.240 |
| PE-P | PE(P-40:5) | 5.862 | 2.389 |
| PE-P | PE(P-38:6) | 8.463 | 2.686 |
| PE-P | PE(P-40:6) | 4.687 | 1.760 |
| PE-P | PE(P-42:6) | 0.339 | 0.108 |
| PE-P | PE(P-40:7) | 7.163 | 2.347 |
| PS | PS(36:1) | 0.142 | 0.025 |
| PS | PS(38:3) | 0.129 | 0.011 |
| PS | PS(38:4) | 0.155 | 0.037 |
| PS | PS(40:6) | 0.126 | 0.007 |
| SM | SM(d30:0) | 0.230 | 0.003 |
| SM | SM(d32:0) | 0.462 | 0.065 |
| SM | SM(d34:0) | 12.263 | 2.494 |
| SM | SM(d35:0) | 0.603 | 0.111 |
| SM | SM(d36:0) | 2.751 | 0.932 |
| SM | SM(d38:0) | 1.968 | 0.410 |
| SM | SM(d40:0) | 3.990 | 1.189 |
| SM | SM(d30:1) | 0.258 | 0.018 |
| SM | SM(d32:1) | 1.778 | 0.432 |
| SM | SM(d33:1) | 1.822 | 0.447 |
| SM | SM(d34:1) | 102.438 | 20.342 |
| SM | SM(d35:1) | 3.804 | 0.995 |
| SM | SM(d36:1) | 18.438 | 6.478 |
| SM | SM(d37:1) | 3.399 | 1.381 |
| SM | SM(d39:1) | 0.650 | 0.166 |
| SM | SM(d41:1) | 11.408 | 2.974 |
| SM | SM(d42:1) | 23.105 | 7.373 |
| SM | SM(d43:1) | 0.363 | 0.076 |
| SM | SM(d32:2) | 0.418 | 0.062 |
| SM | SM(d34:2) | 8.017 | 1.738 |
| SM | SM(d35:2) | 0.434 | 0.064 |
| SM | SM(d36:2) | 8.940 | 2.840 |
| SM | SM(d38:2) | 7.668 | 1.832 |
| SM | SM(d39:2) | 0.813 | 0.282 |
| SM | SM(d40:2) | 2.373 | 0.874 |
| SM | SM(d41:2) | 1.967 | 0.630 |
| SM | SM(d44:2) | 0.476 | 0.109 |
| SM | SM(d36:3) | 0.528 | 0.085 |
| SM | SM(d38:3) | 0.394 | 0.068 |
| SM | SM(d40:3) | 1.547 | 0.448 |
| SM | SM(d41:3) | 0.827 | 0.175 |
| SM | SM(d43:3) | 0.411 | 0.054 |
| SM | SM(d42:5) | 1.644 | 0.451 |
| TG | TG(36:0)_12:0 | 0.024 | 0.122 |
| TG | TG(38:0)_8:0 | 0.005 | 0.068 |
| TG | TG(38:0)_12:0 | 0.049 | 0.265 |
| TG | TG(40:0)_16:0 | 0.055 | 0.223 |
| TG | TG(40:0)_14:0 | 0.061 | 0.274 |
| TG | TG(42:0)_16:0 | 0.300 | 1.144 |
| TG | TG(44:0)_16:0 | 1.152 | 4.795 |
| TG | TG(44:0)_18:0 | 0.054 | 0.182 |
| TG | TG(44:0)_12:0 | 0.460 | 2.437 |
| TG | TG(45:0)_16:0 | 0.344 | 0.250 |
| TG | TG(46:0)_16:0 | 2.125 | 3.939 |
| TG | TG(47:0)_16:0 | 0.244 | 0.339 |
| TG | TG(48:0)_16:0 | 8.957 | 8.837 |
| TG | TG(48:0)_18:0 | 1.168 | 1.786 |
| TG | TG(49:0)_16:0 | 0.340 | 0.504 |
| TG | TG(49:0)_18:0 | 0.172 | 0.212 |
| TG | TG(50:0)_16:0 | 33.504 | 21.782 |
| TG | TG(51:0)_17:0 | 0.038 | 0.042 |
| TG | TG(51:0)_18:0 | 0.136 | 0.159 |
| TG | TG(52:0)_20:0 | 0.335 | 0.357 |
| TG | TG(52:0)_22:0 | 0.022 | 0.040 |
| TG | TG(52:0)_18:0 | 9.331 | 3.510 |
| TG | TG(53:0)_18:0 | 0.039 | 0.031 |
| TG | TG(54:0)_18:0 | 0.823 | 0.177 |
| TG | TG(54:0)_16:0 | 0.123 | 0.150 |
| TG | TG(56:0)_18:0 | 0.033 | 0.034 |
| TG | TG(56:0)_24:0 | 0.014 | 0.019 |
| TG | TG(58:0)_18:0 | 0.007 | 0.015 |
| TG | TG(40:1)_16:0 | 0.049 | 0.123 |
| TG | TG(40:1)_14:0 | 0.024 | 0.122 |
| TG | TG(40:1)_10:0 | 0.025 | 0.142 |
| TG | TG(42:1)_18:1 | 0.117 | 0.457 |
| TG | TG(42:1)_14:0 | 0.063 | 0.229 |
| TG | TG(42:1)_10:0 | 0.057 | 0.197 |
| TG | TG(44:1)_16:1 | 0.282 | 0.928 |
| TG | TG(44:1)_10:0 | 0.283 | 0.709 |
| TG | TG(44:1)_14:0 | 0.386 | 1.048 |
| TG | TG(46:1)_18:1 | 1.795 | 4.232 |
| TG | TG(46:1)_14:0 | 2.344 | 3.804 |
| TG | TG(47:1)_16:0 | 0.491 | 0.528 |
| TG | TG(47:1)_18:1 | 0.321 | 0.398 |
| TG | TG(48:1)_16:0 | 12.250 | 13.798 |
| TG | TG(48:1)_18:1 | 14.506 | 15.058 |
| TG | TG(49:1)_18:1 | 1.103 | 1.066 |
| TG | TG(50:1)_16:0 | 76.872 | 46.614 |
| TG | TG(51:1)_18:1 | 1.319 | 1.208 |
| TG | TG(52:1)_16:0 | 48.028 | 36.244 |
| TG | TG(52:1)_20:0 | 0.149 | 0.166 |
| TG | TG(53:1)_18:0 | 0.239 | 0.255 |
| TG | TG(53:1)_19:1 | 0.033 | 0.044 |
| TG | TG(54:1)_20:1 | 0.279 | 0.407 |
| TG | TG(54:1)_18:0 | 3.157 | 3.382 |
| TG | TG(54:1)_20:0 | 0.950 | 0.999 |
| TG | TG(56:1)_20:0 | 0.049 | 0.074 |
| TG | TG(56:1)_18:1 | 0.162 | 0.263 |
| TG | TG(57:1)_18:1 | 0.017 | 0.020 |
| TG | TG(58:1)_18:1 | 0.077 | 0.115 |
| TG | TG(58:1)_22:0 | 0.019 | 0.041 |
| TG | TG(60:1)_18:1 | 0.015 | 0.024 |
| TG | TG(60:1)_18:0 | 0.008 | 0.017 |
| TG | TG(42:2)_18:2 | 0.057 | 0.270 |
| TG | TG(44:2)_16:1 | 0.088 | 0.172 |
| TG | TG(44:2)_16:0 | 0.170 | 0.424 |
| TG | TG(44:2)_14:0 | 0.077 | 0.244 |
| TG | TG(46:2)_16:0 | 0.722 | 1.840 |
| TG | TG(46:2)_18:2 | 0.774 | 1.693 |
| TG | TG(46:2)_18:1 | 0.479 | 0.826 |
| TG | TG(47:2)_18:2 | 0.162 | 0.199 |
| TG | TG(47:2)_15:0 | 0.171 | 0.176 |
| TG | TG(47:2)_14:0 | 0.140 | 0.163 |
| TG | TG(48:2)_16:0 | 12.088 | 13.056 |
| TG | TG(48:2)_18:2 | 9.017 | 8.562 |
| TG | TG(48:2)_18:1 | 7.097 | 8.648 |
| TG | TG(49:2)_17:1 | 0.510 | 0.570 |
| TG | TG(49:2)_18:2 | 0.925 | 0.939 |
| TG | TG(49:2)_15:0 | 1.066 | 0.983 |
| TG | TG(50:2)_16:0 | 119.998 | 69.293 |
| TG | TG(50:2)_14:0 | 15.389 | 11.567 |
| TG | TG(51:2)_18:1 | 7.712 | 6.146 |
| TG | TG(51:2)_15:0 | 2.337 | 1.767 |
| TG | TG(52:2)_18:1 | 241.763 | 88.947 |
| TG | TG(52:2)_18:0 | 22.433 | 16.090 |
| TG | TG(52:2)_14:0 | 0.201 | 0.173 |
| TG | TG(52:2)_20:1 | 0.632 | 0.476 |
| TG | TG(53:2)_17:0 | 2.845 | 1.688 |
| TG | TG(53:2)_16:0 | 1.023 | 0.615 |
| TG | TG(54:2)_18:0 | 28.644 | 16.878 |
| TG | TG(54:2)_16:0 | 4.119 | 3.186 |
| TG | TG(54:2)_20:1 | 4.248 | 3.274 |
| TG | TG(54:2)_22:1 | 0.063 | 0.102 |
| TG | TG(55:2)_18:1 | 0.144 | 0.121 |
| TG | TG(55:2)_20:1 | 0.032 | 0.030 |
| TG | TG(56:2)_20:1 | 0.311 | 0.373 |
| TG | TG(56:2)_20:0 | 0.352 | 0.513 |
| TG | TG(56:2)_22:1 | 0.164 | 0.357 |
| TG | TG(57:2)_18:1 | 0.046 | 0.060 |
| TG | TG(58:2)_22:0 | 0.082 | 0.173 |
| TG | TG(58:2)_18:0 | 0.017 | 0.068 |
| TG | TG(58:2)_16:0 | 0.051 | 0.105 |
| TG | TG(59:2)_18:1 | 0.008 | 0.011 |
| TG | TG(60:2)_24:0 | 0.029 | 0.050 |
| TG | TG(62:2)_18:1 | 0.008 | 0.015 |
| TG | TG(44:3)_16:1 | 0.020 | 0.058 |
| TG | TG(44:3)_18:2 | 0.047 | 0.175 |
| TG | TG(44:3)_16:0 | 0.028 | 0.055 |
| TG | TG(46:3)_18:2 | 0.241 | 0.363 |
| TG | TG(46:3)_18:1 | 0.119 | 0.227 |
| TG | TG(48:3)_18:1 | 1.459 | 1.756 |
| TG | TG(48:3)_18:2 | 3.519 | 3.810 |
| TG | TG(48:3)_16:0 | 1.383 | 1.830 |
| TG | TG(49:3)_16:1 | 0.261 | 0.233 |
| TG | TG(49:3)_18:2 | 0.353 | 0.322 |
| TG | TG(49:3)_14:0 | 0.085 | 0.097 |
| TG | TG(50:3)_18:1 | 20.150 | 14.008 |
| TG | TG(50:3)_18:2 | 42.896 | 26.125 |
| TG | TG(51:3)_16:1 | 0.816 | 0.653 |
| TG | TG(51:3)_15:0 | 2.649 | 1.926 |
| TG | TG(51:3)_16:0 | 1.703 | 1.363 |
| TG | TG(52:3)_16:1 | 37.507 | 20.340 |
| TG | TG(52:3)_16:0 | 303.490 | 103.288 |
| TG | TG(52:3)_14:0 | 0.322 | 0.234 |
| TG | TG(53:3)_18:1 | 3.319 | 2.205 |
| TG | TG(53:3)_17:0 | 2.629 | 1.730 |
| TG | TG(54:3)_18:1 | 67.556 | 33.039 |
| TG | TG(54:3)_18:0 | 34.553 | 17.772 |
| TG | TG(54:3)_16:0 | 8.184 | 4.435 |
| TG | TG(55:3)_20:1 | 0.056 | 0.066 |
| TG | TG(55:3)_18:1 | 0.559 | 0.363 |
| TG | TG(56:3)_18:2 | 1.497 | 1.341 |
| TG | TG(56:3)_20:1 | 1.144 | 1.574 |
| TG | TG(56:3)_22:1 | 0.275 | 0.429 |
| TG | TG(56:3)_18:0 | 1.075 | 0.668 |
| TG | TG(58:3)_22:1 | 0.026 | 0.039 |
| TG | TG(58:3)_18:2 | 0.506 | 0.788 |
| TG | TG(60:3)_18:1 | 0.054 | 0.090 |
| TG | TG(60:3)_24:0 | 0.043 | 0.074 |
| TG | TG(44:4)_18:2 | 0.012 | 0.075 |
| TG | TG(46:4)_18:1 | 0.016 | 0.036 |
| TG | TG(48:4)_16:2 | 0.064 | 0.076 |
| TG | TG(48:4)_14:1 | 0.144 | 0.173 |
| TG | TG(48:4)_12:0 | 0.391 | 0.435 |
| TG | TG(48:4)_18:1 | 0.121 | 0.133 |
| TG | TG(48:4)_16:1 | 0.206 | 0.264 |
| TG | TG(50:4)_14:0 | 5.494 | 4.770 |
| TG | TG(50:4)_18:2 | 10.550 | 8.554 |
| TG | TG(50:4)_20:4 | 0.485 | 0.776 |
| TG | TG(50:4)_18:3 | 3.476 | 4.362 |
| TG | TG(51:4)_18:2 | 0.724 | 0.563 |
| TG | TG(51:4)_16:1 | 0.219 | 0.190 |
| TG | TG(52:4)_18:2 | 245.964 | 110.828 |
| TG | TG(52:4)_20:4 | 4.205 | 4.596 |
| TG | TG(52:4)_16:0 | 139.862 | 65.570 |
| TG | TG(53:4)_18:1 | 1.868 | 1.468 |
| TG | TG(53:4)_19:2 | 0.057 | 0.044 |
| TG | TG(54:4)_18:1 | 87.033 | 38.763 |
| TG | TG(54:4)_22:3 | 0.038 | 0.044 |
| TG | TG(54:4)_18:0 | 13.674 | 9.142 |
| TG | TG(55:4)_18:1 | 0.257 | 0.195 |
| TG | TG(56:4)_18:1 | 3.685 | 2.197 |
| TG | TG(56:4)_18:0 | 1.100 | 0.675 |
| TG | TG(56:4)_16:1 | 0.028 | 0.039 |
| TG | TG(56:4)_18:2 | 1.235 | 1.013 |
| TG | TG(58:4)_22:1 | 0.171 | 0.405 |
| TG | TG(58:4)_22:2 | 0.053 | 0.047 |
| TG | TG(60:4)_18:1 | 0.065 | 0.149 |
| TG | TG(48:5)_18:2 | 0.055 | 0.058 |
| TG | TG(50:5)_16:1 | 0.091 | 0.109 |
| TG | TG(50:5)_18:2 | 0.567 | 0.549 |
| TG | TG(50:5)_14:1 | 0.202 | 0.172 |
| TG | TG(50:5)_16:0 | 0.162 | 0.219 |
| TG | TG(50:5)_20:4 | 0.145 | 0.246 |
| TG | TG(51:5)_18:2 | 0.137 | 0.138 |
| TG | TG(51:5)_20:4 | 0.020 | 0.037 |
| TG | TG(51:5)_18:3 | 0.103 | 0.125 |
| TG | TG(52:5)_18:2 | 7.348 | 5.510 |
| TG | TG(52:5)_16:0 | 10.515 | 9.645 |
| TG | TG(52:5)_18:3 | 14.906 | 13.539 |
| TG | TG(52:5)_20:4 | 2.548 | 3.301 |
| TG | TG(52:5)_22:5 | 0.200 | 0.166 |
| TG | TG(53:5)_16:0 | 0.068 | 0.095 |
| TG | TG(53:5)_18:2 | 0.386 | 0.335 |
| TG | TG(53:5)_20:4 | 0.264 | 0.260 |
| TG | TG(54:5)_18:2 | 48.065 | 27.822 |
| TG | TG(54:5)_20:4 | 17.761 | 14.070 |
| TG | TG(54:5)_16:1 | 0.895 | 0.643 |
| TG | TG(55:5)_20:4 | 0.197 | 0.137 |
| TG | TG(55:5)_18:2 | 0.084 | 0.069 |
| TG | TG(56:5)_18:2 | 1.959 | 1.320 |
| TG | TG(56:5)_20:2 | 1.487 | 0.879 |
| TG | TG(56:5)_22:4 | 2.377 | 1.782 |
| TG | TG(56:5)_20:4 | 8.362 | 3.815 |
| TG | TG(56:5)_20:1 | 0.708 | 0.614 |
| TG | TG(58:5)_18:2 | 0.060 | 0.096 |
| TG | TG(58:5)_22:2 | 0.035 | 0.031 |
| TG | TG(58:5)_22:4 | 0.161 | 0.119 |
| TG | TG(58:5)_24:4 | 0.078 | 0.076 |
| TG | TG(60:5)_18:2 | 0.034 | 0.047 |
| TG | TG(50:6)_18:2 | 0.054 | 0.063 |
| TG | TG(50:6)_14:0 | 0.029 | 0.058 |
| TG | TG(50:6)_22:6 | 0.056 | 0.144 |
| TG | TG(52:6)_16:0 | 0.537 | 0.812 |
| TG | TG(52:6)_18:2 | 1.208 | 1.172 |
| TG | TG(52:6)_18:4 | 0.474 | 0.768 |
| TG | TG(53:6)_17:1 | 0.073 | 0.096 |
| TG | TG(54:6)_18:1 | 9.058 | 9.273 |
| TG | TG(54:6)_20:4 | 12.635 | 9.479 |
| TG | TG(54:6)_16:1 | 0.861 | 0.745 |
| TG | TG(54:6)_22:6 | 3.174 | 3.735 |
| TG | TG(55:6)_18:1 | 0.114 | 0.122 |
| TG | TG(55:6)_16:0 | 0.116 | 0.148 |
| TG | TG(56:6)_22:5 | 2.895 | 2.474 |
| TG | TG(56:6)_20:4 | 3.295 | 1.483 |
| TG | TG(56:6)_22:6 | 1.708 | 1.663 |
| TG | TG(56:6)_18:2 | 1.089 | 0.770 |
| TG | TG(58:6)_22:4 | 0.353 | 0.229 |
| TG | TG(58:6)_22:6 | 0.188 | 0.236 |
| TG | TG(58:6)_18:0 | 0.294 | 0.194 |
| TG | TG(52:7)_18:3 | 0.128 | 0.196 |
| TG | TG(52:7)_20:5 | 0.138 | 0.223 |
| TG | TG(54:7)_18:2 | 2.337 | 2.918 |
| TG | TG(54:7)_20:5 | 3.186 | 4.173 |
| TG | TG(54:7)_22:6 | 3.248 | 4.042 |
| TG | TG(54:7)_18:4 | 0.408 | 0.519 |
| TG | TG(55:7)_22:6 | 0.298 | 0.388 |
| TG | TG(55:7)_20:5 | 0.057 | 0.123 |
| TG | TG(56:7)_20:4 | 5.764 | 3.375 |
| TG | TG(56:7)_22:6 | 16.111 | 15.731 |
| TG | TG(56:7)_16:1 | 0.157 | 0.139 |
| TG | TG(57:7)_22:6 | 0.182 | 0.208 |
| TG | TG(58:7)_22:6 | 1.770 | 2.235 |
| TG | TG(58:7)_22:5 | 0.884 | 0.477 |
| TG | TG(58:7)_22:4 | 0.392 | 0.275 |
| TG | TG(58:7)_16:0 | 0.252 | 0.200 |
| TG | TG(54:8)_18:2 | 0.302 | 0.432 |
| TG | TG(54:8)_22:6 | 0.532 | 0.704 |
| TG | TG(56:8)_22:6 | 11.808 | 13.249 |
| TG | TG(56:8)_16:1 | 0.331 | 0.394 |
| TG | TG(56:8)_24:6 | 2.033 | 2.243 |
| TG | TG(57:8)_22:6 | 0.231 | 0.317 |
| TG | TG(58:8)_22:5 | 1.108 | 0.646 |
| TG | TG(58:8)_18:1 | 0.676 | 0.702 |
| TG | TG(58:8)_22:6 | 3.293 | 3.294 |
| TG | TG(58:8)_22:4 | 0.142 | 0.112 |
| TG | TG(58:8)_24:6 | 0.178 | 0.156 |
| TG | TG(60:8)_22:6 | 0.083 | 0.151 |
| TG | TG(60:8)_18:1 | 0.058 | 0.055 |
| TG | TG(54:9)_18:3 | 0.042 | 0.093 |
| TG | TG(54:9)_16:0 | 0.063 | 0.091 |
| TG | TG(54:9)_22:6 | 0.047 | 0.077 |
| TG | TG(56:9)_22:6 | 1.485 | 1.940 |
| TG | TG(56:9)_20:4 | 0.368 | 0.356 |
| TG | TG(58:9)_22:6 | 4.501 | 5.477 |
| TG | TG(58:9)_20:4 | 0.648 | 0.564 |
| TG | TG(58:9)_22:5 | 0.670 | 0.512 |
| TG | TG(60:9)_22:6 | 0.103 | 0.123 |
| TG | TG(56:10)_18:3 | 0.037 | 0.056 |
| TG | TG(56:10)_20:5 | 0.090 | 0.154 |
| TG | TG(58:10)_22:6 | 1.176 | 1.471 |
| TG | TG(58:10)_18:2 | 0.381 | 0.440 |
| TG | TG(58:10)_20:4 | 0.336 | 0.295 |
| TG | TG(60:10)_22:6 | 0.254 | 0.284 |
| TG | TG(58:11)_22:6 | 0.206 | 0.289 |
| TG | TG(58:11)_20:5 | 0.080 | 0.147 |
| TG | TG(60:11)_20:4 | 0.176 | 0.170 |
| TG | TG(58:12)_20:5 | 0.048 | 0.119 |
| TG | TG(60:12)_22:6 | 0.226 | 0.450 |
| TG | TG(60:13)_22:6 | 0.095 | 0.186 |
| TG | TG(62:13)_22:6 | 0.178 | 0.386 |
| Abbreviations: BA, bile acid; FFA, free fatty acid; LPG, lysophosphatidylglycerol; LPI, lysophosphatidylinositol; LPS, lysophosphatidylserine; PI, phosphatidylinositol; CerP, ceramide-1-phosphate; CAR, acylcarnitine; Cho, cholesterol; CE, cholesteryl ester; SPH, sphingosine; Cer, ceramide; CoQ, coenzyme Q; DG, diacylglycerol; HerCer, hexosylceramide; LPC, lysophophatidylcholine; LPC-O, alkyl-lysophophatidylcholine; LPE, lysophosphatidylethanolamine; LPE-P, alkenyl-lysophosphatidylethanolamine; PC, phosphatidylcholine; PC-O, alkylglycerophosphocholine; PE, phosphatidylethanolamine; PE-P, alkenylglycerophosphoethanolamine; PS, phosphatidylserine; SM, sphingomyelin; TG, triacylglycerol. | | | |

**Supplementary Table 4. Baseline characteristics of patients in the training set and validation set.**

| Variable | Training set (n=107)  mean$\boldsymbol{\pm}$SD or N (%) | Validation set (n=72)  mean$\boldsymbol{\pm}$SD or N (%) | *P* value^a^ |
| --- | --- | --- | --- |
| Age (yr) | 45.4 ± 12.0 | 46.1 ± 12.3 | 0.702 |
| Sex |  |  | 0.334 |
| Female | 32 (29.9) | 16 (22.2) |  |
| Male | 75 (70.1) | 56 (77.8) |  |
| Tumor stage |  |  | 0.539 |
| T1 | 6 (5.6) | 2 (2.8) |  |
| T2 | 8 (7.5) | 7 (9.7) |  |
| T3 | 72 (67.3) | 44 (61.1) |  |
| T4 | 21 (19.6) | 19 (26.4) |  |
| Node stage |  |  | 0.464 |
| N0 | 12 (11.2) | 6 (8.3) |  |
| N1 | 50 (46.7) | 27 (37.5) |  |
| N2 | 26 (24.3) | 23 (31.9) |  |
| N3 | 19 (17.8) | 16 (22.2) |  |
| Clinical stage |  |  | 0.212 |
| Ⅲ | 72 (67.3) | 41 (56.9) |  |
| Ⅳ | 35 (32.7) | 31 (43.1) |  |
| BMI (kg/m2) | 21.6 ± 1.5 | 21.5 ± 1.5 | 0.775 |
| CHO (mmol/L) | 4.8 (0.9) | 5.1 (1.0) | 0.046 |
| TG (mmol/L) | 1.2 (0.6) | 1.4 (0.9) | 0.146 |
| HDL (mmol/L) | 1.3 (0.3) | 1.3 (0.3) | 0.754 |
| LDL (mmol/L) | 3.0 (0.8) | 3.1 (0.9) | 0.176 |
| WBC (109/L) | 7.0 (1.8) | 7.1 (2.1) | 0.702 |
| Neutrophil (109/L) | 4.4 (1.5) | 4.6 (1.7) | 0.574 |
| Lymphocyte (109/L) | 1.8 (0.6) | 1.8 (0.6) | 0.866 |
| CRP (mg/L) | 3.8 (6.7) | 4.3 (7.3) | 0.627 |
| LDH (U/L) | 185.0 (62.7) | 183.2 (44.5) | 0.820 |
| EBV DNA (copies/ml) |  |  | 0.030 |
| <1000 | 37 (34.6) | 35 (48.6) |  |
| 1000-9999 | 42 (39.3) | 14 (19.4) |  |
| 10000-99999 | 20 (18.7) | 19 (26.4) |  |
| ≥100000 | 8 (7.5) | 4 (5.6) |  |
| Treatment |  |  | 0.521 |
| CCRT | 45 (42.1) | 26 (36.1) |  |
| ICT + CCRT | 62 (57.9) | 46 (63.9) |  |
| Target therapy^b^ | 16 (15.0) | 11 (15.3) | 0.878 |
| Abbreviations: BMI, body mass index; CHO, cholesterol; TG, triglyceride; HDL, high-density lipoprotein cholesterol; LDL, low-density lipoprotein cholesterol; WBC, white blood cell; CRP, C-reactive protein; LDH, lactate dehydrogenase; EBV, Epstein-Barr virus; CCRT, concurrent chemoradiotherapy; ICT, induction chemotherapy.  ^a^*P* values were calculated by the Chi-square test for categorical variables and the Student's T test for continuous variables.  ^b^Target therapy was defined as treatment with Cetuximab or Nimotuzumab. | | | |

**Supplementary Table 5. Associations of lipid species with** **distant metastasis-free survival (DMFS) of Nasopharyngeal carcinoma (NPC) by univariate Cox regression analysis. Only the lipid species with P value<0.05 was demonstrated in the table.**

| Lipid specie | Hazard ratio^1^ | 95% CI | *P* value^a^ |
| --- | --- | --- | --- |
| Cer(d18:1/22:0) | 1.66 | 1.18-2.33 | 0.003 |
| FFA(20:3) | 0.41 | 0.22-0.76 | 0.005 |
| CAR(14:0) | 0.38 | 0.20-0.75 | 0.005 |
| 6,7-DKLCA | 1.53 | 1.14-2.06 | 0.005 |
| PC(17:1_18:1) | 1.55 | 1.13-2.13 | 0.007 |
| Hex2Cer(d18:1/16:0) | 0.53 | 0.33-0.85 | 0.009 |
| PGE2 | 1.32 | 1.06-1.66 | 0.015 |
| FFA(22:4) | 0.45 | 0.24-0.85 | 0.015 |
| FFA(20:4) | 0.47 | 0.26-0.87 | 0.017 |
| LPI(18:2) | 1.40 | 1.06-1.85 | 0.017 |
| FFA(18:2) | 0.52 | 0.30-0.90 | 0.020 |
| CAR(12:1) | 0.43 | 0.21-0.88 | 0.021 |
| FFA(22:1) | 0.50 | 0.28-0.90 | 0.022 |
| CAR(14:1-OH) | 0.49 | 0.26-0.90 | 0.022 |
| CAR(10:0) | 0.22 | 0.06-0.81 | 0.022 |
| FFA(14:0) | 0.33 | 0.13-0.86 | 0.024 |
| CAR(12:1-OH) | 0.55 | 0.32-0.93 | 0.025 |
| CAR12-OH | 0.53 | 0.31-0.93 | 0.026 |
| CAR(12:0) | 0.45 | 0.22-0.92 | 0.028 |
| DG(16:0_20:4) | 1.21 | 1.02-1.44 | 0.029 |
| FFA(18:3) | 0.48 | 0.25-0.93 | 0.029 |
| LPI(18:1) | 1.38 | 1.03-1.85 | 0.030 |
| FFA(18:0) | 0.60 | 0.37-0.95 | 0.031 |
| FFA(17:1) | 0.52 | 0.29-0.95 | 0.032 |
| FFA(16:0) | 0.57 | 0.34-0.96 | 0.033 |
| FFA(20:2) | 0.57 | 0.34-0.96 | 0.033 |
| LPI(16:0) | 1.41 | 1.03-1.95 | 0.034 |
| FFA(18:1) | 0.58 | 0.35-0.96 | 0.035 |
| FFA(20:1) | 0.54 | 0.30-0.97 | 0.040 |
| LPE(P-16:0/0:0) | 1.41 | 1.02-1.96 | 0.040 |
| UCA | 0.71 | 0.51-0.99 | 0.041 |
| LPC(17:0/0:0) | 1.40 | 1.01-1.92 | 0.042 |
| HexCer(d18:1/24:1) | 0.60 | 0.37-0.98 | 0.042 |
| FFA(22:5) | 0.56 | 0.31-0.98 | 0.043 |
| CAR(14:2) | 0.43 | 0.18-0.98 | 0.044 |
| LPC(O-20:2/0:0) | 0.67 | 0.45-0.99 | 0.045 |
| PC(34:2) | 1.44 | 1.01-2.05 | 0.046 |
| Hex2Cer(d16:1/16:0) | 0.66 | 0.43-1.00 | 0.048 |
| HexCer(d18:2/24:1) | 0.63 | 0.40-1.00 | 0.048 |
| CAR(10:1) | 0.41 | 0.17-1.00 | 0.049 |
| ^a^Hazard ratios and *P* values were calculated by univariate Cox regression analysis after adjustment for covariates including age, sex, BMI, T stage, N stage, clinical stage, EBV DNA, CHO, TG, HDL, and LDL. For univariate Cox regression, lipid data were scaled by the standard deviation and mean centred within the training set. Abbreviations: Cer, ceramide; FFA, free fatty acid; CAR, acylcarnitine; DKLCA, diketolithocholic acid; HexCer, hexosylceramide; PG, prostaglandin; LPI, lysophosphatidylinositol; DG, diacylglycerol; LPE, lysophosphatidylethanolamine; UCA, Ursocholic acid; LPC, lysophophatidylcholine; PC, phosphatidylcholine. | | | |

**Supplementary Table 6. C-index of the models based on clinical biomarkers with / without lipid biomarkers in predicting DMFS, PFS, and OS.**

| **Clinical outcome** | **Training set** | | **Validation set** | |
| --- | --- | --- | --- | --- |
|  | **C-index** | **95%CI** | **C-index** | **95%CI** |
| DMFS |  |  |  |  |
| Model based on lipid and clinical biomarkers | 0.764 | 0.682-0.846 | 0.760 | 0.649-0.871 |
| Model based on clinical biomarkers | 0.718 | 0.591-0.845 | 0.672 | 0.511-0.833 |
| PFS |  |  |  |  |
| Model based on lipid and clinical biomarkers | 0.753 | 0.656-0.850 | 0.752 | 0.651-0.853 |
| Model based on clinical biomarkers | 0.692 | 0.596-0.788 | 0.668 | 0.557-0.779 |
| OS |  |  |  |  |
| Model based on lipid and clinical biomarkers | 0.774 | 0.613-0.935 | 0.740 | 0.587-0.893 |
| Model based on clinical biomarkers | 0.695 | 0.549-0.784 | 0.664 | 0.391-0.839 |
| Abbreviations: DMFS, distant metastasis-free survival; PFS, progression-free survival; OS, overall survival; C-index, concordance index; CI, confidence interval. | | | | |

**Supplementary Table 7. Number of events in the high-risk and low-risk groups.**

| **Number of events** | **Training set**  **(n=107)** | | **Validation set**  **(n=72)** | |
| --- | --- | --- | --- | --- |
|  | **Low-risk** | **High-risk** | **Low-risk** | **High-risk** |
| Distant metastasis | 1 of 53 | 21 of 54 | 2 of 37 | 15 of 35 |
| Progression | 8 of 68 | 22 of 39 | 6 of 43 | 18 of 29 |
| Death | 5 of 87 | 5 of 20 | 2 of 51 | 5 of 21 |

| **5-year survival rate,**  **% (95%CI)** | **Training set**  **(n=107)** | | **Validation set**  **(n=72)** | |
| --- | --- | --- | --- | --- |
|  | **Low-risk** | **High-risk** | **Low-risk** | **High-risk** |
| DMFS | 98.1  (94.5-100) | 60.6  (48.8-75.3) | 94.6  (87.6-100) | 57.0  (42.7-76.1) |
| PFS | 97.1  (93.1-100) | 79.5  (67.8-93.2) | 86.0  (76.3-97.1) | 37.9  (23.8-60.4) |
| OS | 94.3  (89.5-99.3) | 75.0  (58.2-96.6) | 96.1  (90.9-100) | 81.0  (65.8-96.8) |
| Abbreviations: DMFS, distant metastasis-free survival; PFS, progression-free survival; OS, overall survival; CI, confidence interval. | | | | |

**Supplementary Table 8. 5-year DMFS, PFS, and OS estimates for the high-risk and low-risk groups.**

**Supplementary Table 9. Baseline characteristics of patients in the high-risk and low-risk groups in predicting DMFS.**

| **Value N (%) or mean ± SD** | **Training set (n=107)** | | | **Validation set (n=72)** | | |
| --- | --- | --- | --- | --- | --- | --- |
|  | **Low-risk (n=53)** | **High-risk (n=54)** | ***P* value^a^** | **Low-risk (n=37)** | **High-risk (n=35)** | ***P* value**^a^ |
| Age (yr) | 43.9 ± 12.4 | 46.9 ± 11.6 | 0.183 | 44.8 ± 13.8 | 47.5 ± 10.6 | 0.361 |
| Sex |  |  | 0.263 |  |  | 1.000 |
| Female | 19 (35.8) | 13 (24.1) |  | 8 (21.6) | 8 (22.9) |  |
| Male | 34 (64.2) | 41 (75.9) |  | 29 (78.4) | 27 (77.1) |  |
| Tumor stage |  |  | <0.001 |  |  | 0.002 |
| T1 | 0 (0) | 6 (11.1) |  | 0 (0) | 2 (5.7) |  |
| T2 | 1 (1.9) | 7 (13.0) |  | 1 (2.7) | 6 (17.1) |  |
| T3 | 47 (88.7) | 25 (46.3) |  | 30 (81.1) | 14 (40.0) |  |
| T4 | 5 (9.4) | 16 (29.6) |  | 6 (16.2) | 13 (37.2) |  |
| Node stage |  |  | <0.001 |  |  | <0.001 |
| N0 | 12 (22.6) | 0 (0) |  | 6 (16.2) | 0 (0) |  |
| N1 | 37 (69.8) | 13 (24.1) |  | 22 (59.5) | 5 (14.3) |  |
| N2 | 4 (7.6) | 22 (40.7) |  | 9 (24.3) | 14 (40.0) |  |
| N3 | 0 (0) | 19 (35.2) |  | 0 (0) | 16 (45.7) |  |
| Clinical stage |  |  | <0.001 |  |  | <0.001 |
| Ⅲ | 48 (90.6) | 24 (44.4) |  | 31 (83.8) | 10 (28.6) |  |
| Ⅳ | 5 (9.4) | 30 (55.6) |  | 6 (16.2) | 25 (71.4) |  |
| EBV DNA (copies/ml) |  |  | 0.186 |  |  | 0.086 |
| <1000 | 20 (37.7) | 17 (31.4) |  | 22 (59.5) | 13 (37.1) |  |
| 1000-9999 | 21 (39.6) | 21 (38.9) |  | 6 (16.2) | 8 (22.9) |  |
| 10000-99999 | 11 (20.8) | 9 (16.7) |  | 9 (24.3) | 10 (28.6) |  |
| ≥100000 | 1 (1.9) | 7 (13.0) |  | 0 (0) | 4 (11.4) |  |
| Treatment |  |  | 0.934 |  |  | 0.003 |
| CCRT | 23 (43.4) | 22 (40.7) |  | 20 (54.1) | 6 (17.1) |  |
| ICT + CCRT | 30 (56.6) | 32 (59.3) |  | 17 (45.9) | 29 (82.9) |  |
| Abbreviations: DMFS, distant metastasis-free survival; EBV, Epstein-Barr virus; CCRT, concurrent chemoradiotherapy; ICT, induction chemotherapy.  ^a^*P* values were calculated by the Chi-square test for categorical variables and the Student's T test for continuous variables. | | | | | | |

**Supplementary Table 10. Baseline characteristics of patients in the high-risk and low-risk groups in predicting PFS.**

| **Value N (%)** | **Training set (n=107)** | | | **Validation set (n=72)** | | |
| --- | --- | --- | --- | --- | --- | --- |
|  | **Low-risk (n=68)** | **High-risk (n=39)** | ***P* value^a^** | **Low-risk (n=43)** | **High-risk (n=29)** | ***P* value**^a^ |
| Age (yr) | 43.9 ± 12.3 | 48.0 ± 11.3 | 0.126 | 45.1 ± 12.3 | 47.6 ± 12.4 | 0.367 |
| Sex |  |  | 0.023 |  |  | 0.585 |
| Female | 26 (38.2) | 6 (15.4) |  | 11 (25.6) | 5 (17.2) |  |
| Male | 42 (61.8) | 33 (84.6) |  | 32 (74.4) | 24 (82.8) |  |
| Tumor stage |  |  | <0.001 |  |  | <0.001 |
| T1 | 3 (4.4) | 3 (7.7) |  | 1 (2.3) | 1 (3.5) |  |
| T2 | 5 (7.4) | 3 (7.7) |  | 5 (11.6) | 2 (6.9) |  |
| T3 | 58 (85.3) | 14 (35.9) |  | 33 (76.8) | 11 (37.9) |  |
| T4 | 2 (2.9) | 19 (48.7) |  | 4 (9.3) | 15 (51.7) |  |
| Node stage |  |  | <0.001 |  |  | 0.002 |
| N0 | 11 (16.2) | 1 (2.4) |  | 6 (14.0) | 0 (0) |  |
| N1 | 39 (57.4) | 11 (28.2) |  | 20 (46.5) | 7 (24.1) |  |
| N2 | 15 (22.0) | 11 (28.2) |  | 13 (30.2) | 10 (34.5) |  |
| N3 | 3 (4.4) | 16 (41.0) |  | 4 (9.3) | 12 (41.4) |  |
| Clinical stage |  |  | <0.001 |  |  | <0.001 |
| Ⅲ | 63 (92.6) | 9 (23.1) |  | 36 (83.7) | 5 (17.2) |  |
| Ⅳ | 5 (7.4) | 30 (76.9) |  | 7 (16.3) | 24 (82.8) |  |
| EBV DNA (copies/ml) |  |  | 0.046 |  |  | 0.005 |
| <1000 | 28 (41.2) | 9 (23.1) |  | 27 (62.8) | 8 (27.6) |  |
| 1000-9999 | 27 (39.7) | 15 (38.4) |  | 7 (16.3) | 7 (24.1) |  |
| 10000-99999 | 11 (16.2) | 9 (23.1) |  | 9 (20.9) | 10 (34.5) |  |
| ≥100000 | 2 (2.9) | 6 (15.4) |  | 0 (0) | 4 (13.8) |  |
| Treatment |  |  | 0.714 |  |  | 0.047 |
| CCRT | 30 (44.1) | 15 (38.5) |  | 20 (46.5) | 6 (20.7) |  |
| ICT + CCRT | 38 (55.9) | 24 (61.5) |  | 23 (53.5) | 23 (79.3) |  |
| Abbreviations: PFS, progression-free survival; EBV, Epstein-Barr virus; CCRT, concurrent chemoradiotherapy; ICT, induction chemotherapy.  ^a^*P* values were calculated by the Chi-square test for categorical variables and the Student's T test for continuous variables. | | | | | | |

**Supplementary Table 11. Baseline characteristics of patients in the high-risk and low-risk groups in predicting OS.**

| **Value N (%)** | **Training set (n=107)** | | | **Validation set (n=72)** | | |
| --- | --- | --- | --- | --- | --- | --- |
|  | **Low-risk (n=87)** | **High-risk (n=20)** | ***P* value^a^** | **Low-risk (n=51)** | **High-risk (n=21)** | ***P* value**^a^ |
| Age (yr) | 43.7 ± 11.4 | 52.9 ± 12.0 | 0.003 | 45.7 ± 12.5 | 47.0 ± 12.1 | 0.673 |
| Sex |  |  | 0.179 |  |  | 1.000 |
| Female | 29 (33.3) | 3 (15.0) |  | 11 (21.6) | 5 (23.8) |  |
| Male | 58 (66.7) | 17 (85.0) |  | 40 (78.4) | 16 (76.2) |  |
| Tumor stage |  |  | <0.001 |  |  | <0.001 |
| T1 | 4 (4.6) | 2 (10.0) |  | 1 (2.0) | 1 (4.8) |  |
| T2 | 8 (9.2) | 0 (0) |  | 6 (11.8) | 1 (4.8) |  |
| T3 | 69 (79.3) | 3 (15.0) |  | 39 (76.4) | 5 (23.8) |  |
| T4 | 6 (6.9) | 15 (75.0) |  | 5 (9.8) | 14 (66.6) |  |
| Node stage |  |  | 0.330 |  |  | 0.263 |
| N0 | 11 (12.6) | 1 (5.0) |  | 6 (11.8) | 0 (0) |  |
| N1 | 40 (46.0) | 10 (50.0) |  | 20 (39.2) | 7 (33.3) |  |
| N2 | 23 (26.5) | 3 (15.0) |  | 16 (31.4) | 7 (33.3) |  |
| N3 | 13 (14.9) | 6 (30.0) |  | 9 (17.6) | 7 (33.4) |  |
| Clinical stage |  |  | <0.001 |  |  | <0.001 |
| Ⅲ | 70 (80.5) | 2 (10.0) |  | 38 (74.5) | 3 (14.3) |  |
| Ⅳ | 17 (19.5) | 18 (90.0) |  | 13 (25.5) | 18 (85.7) |  |
| EBV DNA (copies/ml) |  |  | 0.070 |  |  | 0.001 |
| <1000 | 33 (37.9) | 4 (20.0) |  | 31 (60.8) | 4 (19.0) |  |
| 1000-9999 | 35 (40.2) | 7 (35.0) |  | 9 (17.6) | 5 (23.8) |  |
| 10000-99999 | 15 (17.3) | 5 (25.0) |  | 11 (21.6) | 8 (38.1) |  |
| ≥100000 | 4 (4.6) | 4 (20.0) |  | 0 (0) | 4 (19.1) |  |
| Treatment |  |  | 0.337 |  |  | 0.559 |
| CCRT | 39 (44.8) | 6 (30.0) |  | 20 (39.2) | 6 (28.6) |  |
| ICT + CCRT | 48 (55.2) | 14 (70.0) |  | 31 (60.8) | 15 (71.4) |  |
| Abbreviations: OS, overall survival; EBV, Epstein-Barr virus; CCRT, concurrent chemoradiotherapy; ICT, induction chemotherapy.  ^a^*P* values were calculated by the Chi-square test for categorical variables and the Student's T test for continuous variables. | | | | | | |

**Supplementary Table 12. Pathway enrichment and topology analysis result of the predictive lipid species.**

| **Pathway Name** | **Match Status** | **Hit lipid species** | ***P* value** | **Holm *p*** | **FDR** | **Impact** |
| --- | --- | --- | --- | --- | --- | --- |
| Sphingolipid metabolism | [2/21](https://www.metaboanalyst.ca/MetaboAnalyst/Secure/pathway/ResultView.xhtml) | C00195, C01290 | 0.005 | 0.030 | 0.017 | 0.270 |
| Biosynthesis of unsaturated fatty acids | [1/36](https://www.metaboanalyst.ca/MetaboAnalyst/Secure/pathway/ResultView.xhtml) | C00157 | 0.006 | 0.030 | 0.017 | 0.000 |
| Glycerophospholipid metabolism | [1/36](https://www.metaboanalyst.ca/MetaboAnalyst/Secure/pathway/ResultView.xhtml) | C03242 | 0.071 | 0.286 | 0.071 | 0.094 |
| Arachidonic acid metabolism | [1/36](https://www.metaboanalyst.ca/MetaboAnalyst/Secure/pathway/ResultView.xhtml) | C00157 | 0.071 | 0.286 | 0.071 | 0.000 |
| Linoleic acid metabolism | [1/5](https://www.metaboanalyst.ca/MetaboAnalyst/Secure/pathway/ResultView.xhtml) | C00157 | 0.071 | 0.286 | 0.071 | 0.000 |
| alpha-Linolenic acid metabolism | [1/13](https://www.metaboanalyst.ca/MetaboAnalyst/Secure/pathway/ResultView.xhtml) | C00157 | 0.071 | 0.286 | 0.071 | 0.000 |
